# Tubulin Post-Translational Modifications: The Elusive Roles of Acetylation

**DOI:** 10.3390/biology12040561

**Published:** 2023-04-06

**Authors:** Bruno Carmona, H. Susana Marinho, Catarina Lopes Matos, Sofia Nolasco, Helena Soares

**Affiliations:** 1 Centro de Química Estrutural, Institute of Molecular Sciences, Faculdade de Ciências, Universidade de Lisboa, Campo Grande, 1749-016 Lisboa, Portugal; bfcarmona@ciencias.ulisboa.pt (B.C.);; 2 Escola Superior de Tecnologia da Saúde de Lisboa, Instituto Politécnico de Lisboa, Av. D. João II, Lote 4.69.01, 1990-096 Lisboa, Portugal; sbnarciso@estesl.ipl.pt; 3 Centro de Química Estrutural, Institute of Molecular Sciences, Departamento de Química e Bioquímica, Faculdade de Ciências, Universidade de Lisboa, Campo Grande, 1749-016 Lisboa, Portugal; hsmarinho@ciencias.ulisboa.pt; 4 CIISA—Centro de Investigação Interdisciplinar em Sanidade Animal, Faculdade de Medicina Veterinária, Universidade de Lisboa, Avenida da Universidade Técnica, 1300-477 Lisboa, Portugal

**Keywords:** acetylation, tubulin, Lys40, microtubules, post-translational modifications, αTAT1, HDAC6, SIRT2, microtubule-associated proteins, microtubule-mechanical properties

## Abstract

**Simple Summary:**

Microtubules (MTs) are dynamic structures that compose part of the cell cytoskeleton. They play important roles in various cellular functions, such as intracellular transport, cell division, and cell movement. MTs are made up of α/β-tubulin heterodimers that present a diversity due to the existence of different isotypes and post-translational modifications (PTMs). One specific PTM, tubulin-acetylation, occurs inside the MT lumen and has been found to enhance MT flexibility and prevent structural damage. This PTM is also associated with cellular responses to stress and various human pathologies. The regulation of enzymes involved in tubulin acetylation and deacetylation is important for maintaining proper cell physiology. While the role of tubulin-acetylation in MT stability remains debatable, it is clear that PTMs contribute to the unique biochemical and biophysical properties of MTs, creating a code that allows for cellular responses to different environmental cues.

**Abstract:**

Microtubules (MTs), dynamic polymers of α/β-tubulin heterodimers found in all eukaryotes, are involved in cytoplasm spatial organization, intracellular transport, cell polarity, migration and division, and in cilia biology. MTs functional diversity depends on the differential expression of distinct tubulin isotypes and is amplified by a vast number of different post-translational modifications (PTMs). The addition/removal of PTMs to α- or β-tubulins is catalyzed by specific enzymes and allows combinatory patterns largely enriching the distinct biochemical and biophysical properties of MTs, creating a code read by distinct proteins, including microtubule-associated proteins (MAPs), which allow cellular responses. This review is focused on tubulin-acetylation, whose cellular roles continue to generate debate. We travel through the experimental data pointing to α-tubulin Lys40 acetylation role as being a MT stabilizer and a typical PTM of long lived MTs, to the most recent data, suggesting that Lys40 acetylation enhances MT flexibility and alters the mechanical properties of MTs, preventing MTs from mechanical aging characterized by structural damage. Additionally, we discuss the regulation of tubulin acetyltransferases/desacetylases and their impacts on cell physiology. Finally, we analyze how changes in MT acetylation levels have been found to be a general response to stress and how they are associated with several human pathologies.

## 1. Introduction

Post-translational modifications are covalent alterations introduced in protein amino acid sequences during or after their synthesis. In most cases, distinct functional groups are covalently bound to specific amino acid residues in the protein’s primary structure by the activity of specific enzymes. In certain cases, these enzymatic modifications consist of the removal/addition of specific amino acid residues in the protein’s primary sequence. In general, these modifications are transitory and can respond to cues and challenges imposed by the cellular environment. Transient PTMs require another group of specific enzymes that catalyze the removal of PTMs. Different or the same types of PTMs can exist in the same protein: this creates the possibility of combinatory patterns that expand the number of distinct primary sequences of a given protein, most probably conferring specific three-dimensional structures with variations that can be associated with distinct functions in the same cell or that can be specific of cell types and tissues. We may envisage that this also constitutes a code that can be read by distinct groups of interactors, modifiers, and modulators of the protein, triggering distinct signalling cascades and responding to cellular challenges. The complexity of this network is increased if we include the regulation of the enzymes that catalyze the addition and removal of the post-translational modification, which can create, in every moment, a balance between different forms of the protein. Therefore, PTMs drastically increase the number of products made available by the expression of the genes in the genome (for 20,359 encoded gene products 192,917 PTMs are estimated, https://www.nextprot.org/about/human-proteome (accessed on 8 February 2023). Additionally, PTMs introduce new levels of regulation that fine-tune the role of each protein type, adjusting this to specific requirements of specific cell types or/and being able to respond to different signals or signals networks. Illustrating this complexity of PTMs that some proteins are subjected are P53 (regulating cellular processes, such as cell cycle arrest, DNA repair, apoptosis, ferroptosis, senescence, or autophagy to promote cell survival or limit the malignant transformation of a cell) [1], histones (affecting processes, such as transcription, recombination, replication, DNA repair, and the modulation of genomic architecture) [2] and tubulins (affecting, for example, the dynamic behavior of MTs and MTs structures assembly, organization, and remodeling) [3].

In this review, we will focus on one of the most elusive post-translational modifications of tubulin- acetylation—which, along with methylation, are the only tubulin post-translational modifications known to occur inside the MT lumen and whose impact on MTs properties/functions has been a matter of controversy since its discovery. Specifically, we intend to explore the current view on tubulin acetylation’s impact on the MTs’ structure, its role in cellular function, how it is regulated, and how alterations in tubulin acetylation patterns affect the physiology of organisms.

### 1.1. The Dynamic Nature of Microtubules

**Figure 1:**
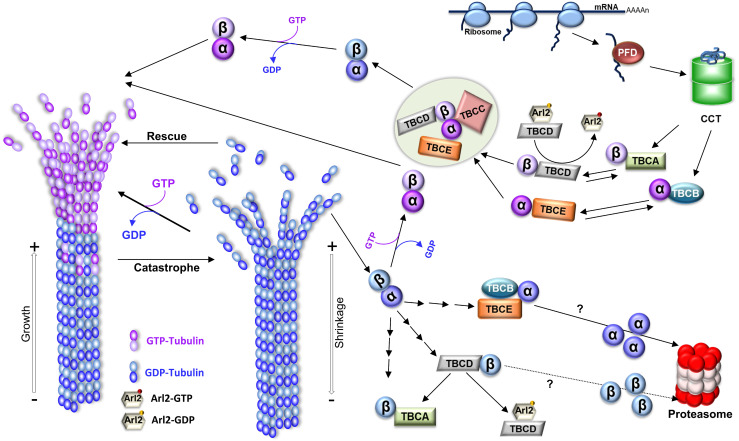
Microtubule structure, dynamics, and tubulin folding and recycling pathways. To originate MTs, tubulin heterodimers interact head-to-tail, arranging in linear protofilaments. The lateral association of the protofilaments, in general, 13 protofilaments, creates the hollow cylindrical structure of the MT with about 25 nm of diameter. Since tubulin heterodimers are oriented inside the MT, this produces a structural polarity; one end of the MT always initiates with α-tubulin, whereas the other terminates with β-tubulin, which is reflected in the dynamic behavior of the polymer with the two ends of a MT displaying different rates of polymerization, a faster-growing end (where β-tubulin is exposed; plus end), and a slower-growing end (where the α-tubulin is ex-posed; minus end). MTs polymerize from soluble tubulin heterodimers bound to guanosine triphosphate (GTP). After incorporation in the polymer, the β-tubulin GTPase activity is activated, GTP is hydrolyzed to guanosine diphosphate (GDP), and the energy from that hydrolysis is assumed to induce conformational changes in tubulin, reducing the stability of the MT lattice (for review [12]). In growing MTs, most heterodimers in the lattice are bound to GDP. However, whenever a MT maintains a cap of GTP tubulin heterodimers at the tip, the entire polymer will be stabilized (the GTP cap model), and the MT grows. On the contrary, if the tip of the MT accumulates a critical number or density of GDP-tubulins, the MT lattice will become unstable, and the MT will transition to a depolymerization state, rapidly shrinking its length (a process designated as “catastrophe”). MTs show stochastic transitions between periods of shrinkage (catastrophe) and growth (rescue) in a process designated “dynamic instability”. A schematic representation of the tubulin folding and native dimer disassembly pathways is also shown. The CCT (cytosolic chaperonin-containing TCP1) captures tubulin folding intermediates, with important native-like domain structures, either directly from ribosomes or from the hetero-hexameric chaperone prefoldin (for review [13]). α- and β-tubulin monomers released from CCT follow different pathways: α-tubulin is captured by cofactor B (TBCB) and β-tubulin by cofactor A (TBCA). Then, cofactors E (TBCE) and D (TBCD) capture α- and β-tubulin, respectively. Additionally, after TBCC binding, a supercomplex is formed. TBCC stimulates GTP hydrolysis by β-tubulin and the consequent release of α/β-tubulin-GDP heterodimers. Upon exchange of GDP by GTP, a functional α/β-tubulin dimer competent to polymerize into a MT is formed. Along with tubulin folding, tubulin cofactors assist tubulin heterodimer assembly/dissociation, as well as tubulin degradation. Tubulin heterodimers released from MTs can be dissociated by cofactors and recycled or degraded. TBCD and TBCE are capable of dissociating the tubulin heterodimer by themselves, but in the case of TBCE, its dissociation activity is highly increased by the presence of TBCB. In this process, the β-tubulin is retained by TBCD, whereas α-tubulin is stabilized by the complex TBCB/TBCE. TBCA mainly receives β-tubulin from the dissociation of pre-existing heterodimers instead of newly synthesized tubulins. Both pathways may lead to the tubulin monomer degradation through the ubiquitin-proteasome system by unknown mechanisms. By recycling the tubulin heterodimers, the TBCE/TBCB+TBCA system is crucial for controlling the critical concentration of free tubulin heterodimers and MT dynamics in the cells [14]. TBCD activity is regulated by Arl2, a small GTP (guanosine triphosphate) binding protein of the Arf family. The figure was inspired on [15,16].

Microtubules are dynamic polymers of α/β-tubulin heterodimers found in all eukaryote cells. These polymers are intrinsically polar, and they stochastically change between phases of growth and shrinkage: a phenomenon known as dynamic instability (see Figure 1). This MT behavior is associated with the requirement of GTP-tubulin heterodimers for MT polymerization and GTP hydrolysis by β-tubulin after polymerization [4]. However, the dynamic instability of MTs cannot explain all features of MT dynamics, such as, for example, the dynamics of aged MT. Thus, more recently, MT tip structures have also been implicated in the mechanisms of tubulin polymerization and dynamic instability (for review [4]). In vivo, MT dynamics depend on the presence of competent tubulin heterodimers, either synthesized de novo or recycled from pre-existing MTs [5]. To support this process, eukaryotic cells have several molecular chaperones, including the cytosolic chaperonin CCT (cytosolic chaperonin-containing TCP1) and its cochaperone prefoldin [6,7], as well as a group of specialized tubulin cofactors (TBCA-E) [8,9]. In addition to supporting tubulin folding, tubulin cofactors also aid in the assembly/disassembly of tubulin heterodimers, as well as their degradation (see Figure 1) [8,9,10,11]. Therefore, tubulin cofactors are essential for preserving the quality of tubulin pools and the recycling/degradation of tubulin heterodimers in vivo.

### 1.2. The Functional Diversity of Microtubules and Microtubule-Based Structures

Microtubules play a myriad of functions in eukaryotes cells and are the components of complex structures, such as the mitotic spindles involved in cell division, centrosome/centrioles that are MT and actin organizing centers, and cilia that can generate motility and have sensory functions coupled with a variety of signaling pathways. The dynamic MT behavior allows MT ends to explore the cell space searching for and binding to intracellular structures (e.g., chromosomes during mitosis) and organelles, such as mitochondria and melanosomes, contributing to their dynamic positioning and organization of the cytoplasm [4,17,18,19]. Additionally, MTs are targeted toward focal adhesions by interacting with actin and intermediate filaments, and their plus ends are then captured and anchored to the cell cortex near these structures [20]. The turnover of focal adhesions is dependent on MTs, although the mechanisms have not been completely elucidated. However, it is well established that MTs are involved in the transport of exocytic vesicles containing cargos that are delivered near focal adhesions and control endocytosis and integrin internalization [21,22]. Consequently, MT networks regulate cellular adhesion to the extracellular matrix and cell migration. Cell migration is also essential in wound healing/tissue repair, tissue renewal, and during immune responses and angiogenesis, and it contributes to metastasis in several types of cancers [23,24,25,26].

In crosstalk with actin and intermediate filaments, the MT cytoskeleton also dynamically organizes the cytoplasm space and is involved in cell shape, which can be quickly remodeled in response to internal and external cues [27]. This is clearly illustrated by different events dependent on MT dynamics occurring during development, such as cell division, cell migration, cell polarization, and differentiation, with implications in cell fate and morphogenesis.

Polarity can be viewed as the asymmetric spatial organization and localization of biomolecules and cellular components (e.g., membrane domains, organelles such as the Golgi apparatus, mitochondria, centrosomes, cilia, and others) that originates structural/functional asymmetries [28,29,30]. The establishment of polarity is important for biological behavior at the individual cell level and for the three-dimensional organization of tissues and organs. For example, epithelial cells are permanently polarized, possessing basolateral surface domains characterized by distinct compositions of proteins and lipids that are established and maintained by tight junctions. During their morphogenesis, MTs undergo a dramatic remodeling changing from an aster organized by the centrosome to a non-centrosomal network that aligns along the apical-basolateral polarity axis [31,32]. Recently, it was shown that these MT arrays are able to bear compressive forces, and the cells are shorter in the absence of these MT-based forces. Moreover, the fact that MTs coupled with adherens junctions through the fat planar cell polarity signalling pathway allows the patterns of these forces to travel through the epithelia [33]. This shows that individual cell MT organization regulates not only cell response to forces, but also coordinates the collective response of cells during tissue morphogenesis [33].

Another example of MT cytoskeleton remodeling assisting morphogenesis is illustrated by platelets’ formation from their precursor cells, the megakaryocytes. These cells are characterized by membrane structures, called the demarcation membrane system, required for the elongation of proplatelet shafts, as well as protrusions that then shed the proplatelets from their tips (for review [34]). The formation of proplatelets requires dynamic remodeling and profound changes in the actin and MT cytoskeleton, such as the continuous growth of MT plus-ends and sliding of adjacent MTs [35]. Platelet size is limited by MT bundling, elastic bending, and actin–myosin–spectrin cortex forces [36].

Cilia biogenesis is also accompanied by cytoskeleton remodeling. During primary cilia assembly, the centrosome leaves its position at the cell center and migrates toward the cell membrane. During this migration, the older centriole (mother centriole) undergoes a complex conversion process into a basal body that finally anchors to the plasma membrane via distal appendages and assembles the MT ciliary axoneme [37]. The centrosome migration is driven by the cytoskeleton remodeling characterized by increased MT nucleation and/or stabilization and bundling, accompanied by a contraction and symmetry breaking of the actin network [38]. Microtubules assemble into a large bundle oriented between the centrosome and the cell’s basal pole, pushing the centrosome toward the apical membrane. The distal appendage protein Cep164 appears to be a critical player in MT cytoskeleton remodeling during centrosome migration [38].

### 1.3. In Vivo Microtubule Dynamics Is Modulated by Microtubule-Associated Proteins

In all of the referred processes, involving MTs, cells show the ability to modulate and explore their dynamic instability and organization by regulating the interaction of these polymers with a large family of motor proteins that produce forces and movement and other MT-associated proteins (MAPs) [39,40]. For example, in cell division, an overall dramatic reorganization of the interphase MT array that culminates with the mitotic spindle requires the combined action of MAPs, including motor proteins [41].

In interphase cells, centrosomes are usually located at the cell center in close association with the nucleus [42]. This localization is dynamic and, together with the nucleus position, constitutes a primary polarity axis for organelle organization in the cytoplasm. One of the important factors acting in the complex landscape of centrosome positioning is the balance of pushing and pulling forces acting on the centrosome generated by MT dynamics, MAPs, including motors (e.g., kinesins and dyneins), as well as specialized cell cortex anchor sites [43,44,45,46,47,48,49]. On the other hand, nucleus positioning also depends on pushing forces [50,51,52]. Consequently, the overall MT organization plays a pivotal role in the spatial distribution of pushing forces [53,54], and it changes in this organization, which will influence the positions of the centrosome and the nucleus.

MAPs are a vast and complex family of distinct proteins that either bind through and stabilize the MT lattice, e.g., Tau, MAP2, and MAP4, or bind to the MTs ends, e.g., the plus tip-binding proteins (+TIPs), EB1 [55], and the CAP-GLY-containing proteins, e.g., the +TIP cytoplasmic linker protein CLIP-170. Plus-end tracking proteins (+TIPs) are specific MAPs that are conserved in all eukaryotes and specifically accumulate at the growing MT plus ends and regulate MTs dynamics. MTs interact with cellular structures, membranes, signaling factors, and the forces exerted in MT arrays [56]. MAPs’ roles have an impact on various critical cellular activities, such as intracellular transport, cell division, polarity establishment, cell motility, and morphogenesis. Among +TIPs, the end-binding protein (EBs) family tracks the growing MTs ends and regulates MT dynamics both directly and by recruiting a variety of other unrelated +TIPs, such as CAP-Gly-containing proteins (CLIP-170, CLIP-115, p150Glued) [57,58]. In vitro, EB1 increases MT nucleation and growth rate and promotes both catastrophes and rescues [55,59,60]. EB proteins possibly bind to the tubulin-GTP (or GDP-Pi) MT cap [61,62,63], and the size of the EB binding region tends to decrease [61] prior to MT transition from growth to depolymerization. Therefore, long binding regions of EB proteins originate protective caps that stabilize the MT [64]. Other +TIPs, e.g., CLASPs, spectraplakins, and APC, which usually act as MT-stabilizing factors, are involved in MT capture and stabilization near the leading edge of migrating cells [65,66,67]. By contrast, the +TIP kinesin-13 family member can remove tubulin subunits by the tip hydrolyzing ATP and promoting catastrophe [68]. Other MAPs can also promote MT depolymerization, such as stathmin, which sequesters tubulin heterodimers, increasing MT catastrophe [69]. Stathmin also binds to protofilaments exposed at the tips of growing MTs and directly promotes catastrophe, at least in part, by interfering with lateral bonding between subunits [70].

Recently, MAPs that stabilize MT dynamic properties (e.g., XMAP215, TPX2, and CAMSAP/Patronin) have been found to contribute to MT nucleation, a role mainly attributed to the γ-tubulin ring complex (for review [71]). MAPs also comprise the MT-severing enzymes, namely, katanin [72], spastin [73,74], and fidgetin [75], which are members of the meiotic subfamily of AAA ATPases [76,77]. These enzymes create internal damage in the MT lattice by active extraction of tubulin heterodimers, causing depolymerization and catastrophe [75,78], and they play important roles, as, for example, in cell division [79], neurogenesis [80,81,82], and cilia biology [83]. More recently, MT-severing enzymes started to emerge as able to amplify MT arrays by promoting the incorporation of GTP-tubulin throughout the MT lattice, as well as by promoting the regrowth of severed MTs, increasing the number and the mass of these polymers [76,84]. Similar behavior was observed for the +TIP CLIP-family members that can function as rescue factors for shrinking MTs [85]. The roles played by MAPs are not limited to the regulation of MTs stability/dynamics and linking with cellular structures, since they are also involved in the control of MT architecture by regulating, for example, MT spacing [86,87,88], cross-talk of MTs with other cytoskeleton filaments (for review [40]), control of MT protofilament numbers (doublecourtin) [89], regulation of motor motility [90], and in the cross-linking of adjacent MTs promoting bundling. For example, the PRC1 protein (MAP65) crosslinks antiparallel MTs [91] that are required for cytokinesis at the end of anaphase at the spindle midzone [92]. The PRC1 protein recruits to this region the Xklp1 kinesin (Xenopus kinesin-4) that guarantees the maintenance of the size of the MTs overlap [92].

More recently, using Cryo-ET, it has been shown that the MT doublets of the motile ciliary axonemes have inner protein structures that periodically bind inside its lumen, creating a sheath (for review [93]). Although the exact role of these MT inner proteins (MIPs) is not yet completely elucidated, they establish a unique architecture of the MT doublet, stabilizing it against strong mechanical forces during cilia beating [93,94]. Additionally, their organization and periodicity limit the MT protofilaments that can be used for the intraflagellar transport (IFT) required for the assembly and maintenance of cilia [95].

In addition, diverse proteins, which can bind to MTs, are involved in signal transduction, protein translation, and metabolism [96]. Finally, it is observed that mechanical forces produced by kinesin and dynein MT motor proteins can remove tubulin dimers from the lattice and destroy MTs [97,98]. Triclin and coworkers (2021) showed the existence of a MT self-repair mechanism where tubulin heterodimer removal can be compensated for the insertion of free tubulin dimers into the MT lattice [98]. It has been proposed that MAPs may play a role in this process, maintaining the MT lattice integrity [40]. The vast functional diversity of MAPs suggests that distinct MT arrays may be regulated by different balances of distinct MAPs and respond to different MAPs networks that can locally originate distinct environments in response to various signals. MT recognition/binding by MAPs may be affected by diverse post-translational modifications (PTMs) of tubulin or by incorporating distinct ratios of different tubulin isotypes [99].

### 1.4. The In Vivo Diversity of Tubulin Pools and Microtubule Functional Diversity

The biochemical diversity of tubulin pools is generated by a combination of different tubulin isotypes encoded by the distinct tubulin gene family members, which may exhibit constitutive, developmental, and tissue-specific expression patterns, as well as by diverse PTMs that tubulin can experience [15,100]. There is growing evidence that specific tubulin isotypes encoded by members of tubulin multigenic families may be required to assemble functional distinct MT structures [101,102]. For example, the product of the β2-gene of *Drosophila* is important for the correct axonemal structure in sperm, and males expressing mutations in this gene are infertile [103,104]. Moreover, in *Caenorhabditis elegans,* α- and β-tubulin (coded by genes *mec-12* and *mec-7*, respectively) are specific for the assembly of MTs, owning to 15 protofilaments that are found in the touch receptor neurons, showing that this specific α-tubulin isotype is required for the assembly of a specific class of neuronal MTs [105,106]. In mammals, the divergent β1-tubulin isotype is expressed exclusively in platelets and megakaryocytes. β1-tubulin has a specialized role in platelet synthesis, structure, and function, and it is required to maintain the high degree of MT bundling and elastic bending necessary for the specialized MT arrays of platelets [36,107].

**Figure 2:**
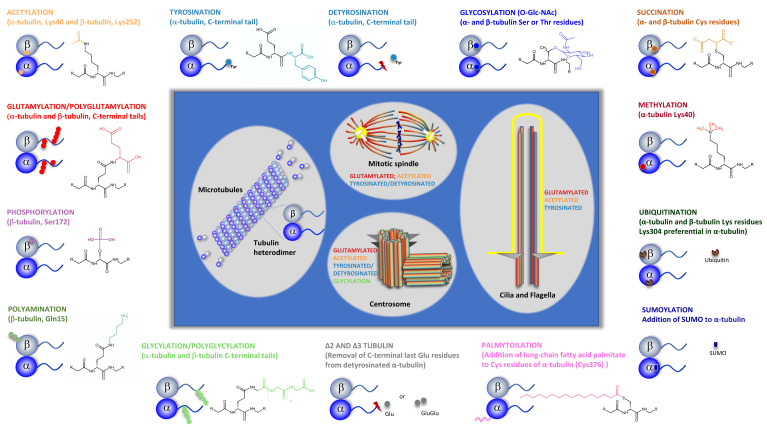
Tubulin post-translational modifications. Specific groups of functionally distinct MT structures and their more common PTMs are highlighted (i.e., centrosome, cilia, and mitotic spindle). Specific tubulins amino acid residues or tubulin specific domains where PTMs occur are indicated. PTMs chemical structure are specified. Tubulin PTMs caused by association with specific proteins, such as SUMO and ubiquitin, as well as those originated by specific proteolysis (detyrosination and Δ2 and Δ3 tubulin) are also shown. For Glutamylation PTM, only the chemical structure corresponding to monoglutamylation is shown.

On the other hand, tubulins present a complex pattern of distinct conserved PTMs (See Figure 2; reviewed in [108,109]), namely: (i) phosphorylation, i.e., the addition of a phosphate group to Ser, Thr, or Tyr residues in α- and β-tubulins, including β-tubulin Ser172, β3-tubulin Ser444, α-tubulin Tyr432, and yet not identified α-tubulin and β-tubulin Tyr residues [110,111,112,113,114,115]; (ii) methylation of α-tubulin at Lys40 [116] and Lys311 and β-tubulin at Lys19 and Lys297 [117]; (iii) palmitoylation, i.e., addition of long-chain fatty acid palmitate to Lys residues with α-tubulin Lys376 being a major modification site [118]; (iv) polyamination, i.e., addition of polyamines to the γ- carboxamide group of Gln side chains in α- and β-tubulin, with β-tubulin Gln15 being the major modification site [119]; (v) tyrosination/detyrosination, i.e., the enzymatic ligation of tyrosine and the enzymatic removal of tyrosine, respectively, which occurs at the α-tubulin C-terminal Tyr residue [120,121,122]; (vi) glycylation and polyglycylation, i.e., the addition of Gly to the γ-carboxy group of Glu side chains and chain elongation by additional addition of Gly residues; multiple Glu residues can be modified in α- and β-tubulin C- terminal tails [123]; (vii) glutamylation and polyglutamylation, i.e., addition of Glu to the γ-carboxy group of Glu side chains and chain elongation by additional addition of Glu residues; multiple Glu residues can be modified in α- and β-tubulin C-terminal tails [124,125,126,127]; (viii) ubiquitination, i.e., the addition of ubiquitin to Lys residues of tubulin, with α-tubulin Lys304 being the major modification site [128,129]; (ix) sumoylation, i.e., covalent conjugation of SUMO (small ubiquitin-related modifier) [130]; (x) Creation of Δ2-tubulin and Δ3-tubulin by removal of C-terminal penultimate Glu residues from detyrosinated α-tubulin [131,132,133] and (xi) acetylation, i.e., the addition of an acetyl group to Lys residues, with at least 12 sites of acetylation in α-tubulin identified by proteomic studies [134,135,136,137,138,139] (for details, see the next sections). Tubulin succination, a modification that occurs when fumarate reacts with cysteine residues to generate S-(2-succino)cysteine [140], and the α- and β-tubulin glycosylation of Ser or Thr residues by the binding of O-linked β-N-acetylglucosamine (O-Glc-NAc)) [136,141], are the two less-studied tubulin PTMs (Figure 2). Although not much is known about succinated tubulin, it seems that this post-translation modification is more abundant in more dynamic MTs [140]. The in vitro addition of O-Glc-NAcylation to α-tubulin, but not to β-tubulin, causes a decrease in the interactions required for dimer assembly and inhibits tubulin polymerization [136,141]. The extensive diversity and complexity of distinct tubulin PTMs led to the proposal that they would create a pattern on the MT surface, which is known as the “tubulin code” [142].

Tubulin PTMs are reversible and regulated and, in the last years, many of the enzymes that catalyze the formation or removal of these modifications have been identified [3,108,143]. Interestingly, the enzymes that catalyze the formation of PTMs seem to have a catalytic preference for MTs over free tubulin as substrates. This preference for MTs is illustrated by the carboxypeptidase catalyzing tubulin detyrosination [144], the specific acetyltransferase α-tubulin acetyltransferase 1 (αTAT1), which catalyzes α-tubulin Lys40 acetylation, as well as tubulin polyglutamylase [145,146]. On the contrary, the enzymes that remove tubulin PTMs, such as deglutamylases, can catalyze deglutamylation on both polymerized and soluble tubulin, whereas human histone deacetylase 6 (HDAC6), the major deacetylase catalyzing the removal of the acetyl group from α-tubulin Lys40, prefers tubulin dimers as substrates [147,148], and tubulin-tyrosine ligase (TTL) exclusively uses tubulin dimers as substrates [149,150]. One exception to these observations is the phosphorylation of β-tubulin Ser172, catalyzed by the cyclin-dependent kinase Cdk1 during the transition from interphase to mitosis and by the MNB/DYRK1a kinase, which regulates MT dynamics in neurons and which occurs mainly at the polymer and inhibits the polymerization of the heterodimers [110,111].

Although it is known that tubulin can undergo a large variety of rapid and reversible PTMs, some of which have been studied in depth, the knowledge about the crosstalk between those modifications is still sketchy. A possible crosstalk between tubulin glycylation and glutamylation has been suggested [151]. These PTMs occur within the same cluster of glutamate residues, which may indicate a possible competition between tubulin glycylation and glutamylation. In *Tetrahymena* and *Drosophila*, mouse loss of glycylation is accompanied by tubulin hyperglutamylation, indicating that both PTMs are possibly regulated together [152,153,154]. Another crosstalk is that between methylation and acetylation, since there is an obvious competition for the same α-tubulin lysine residue (Lys40) in MTs, which can undergo either acetylation or methylation catalyzed by the methyltransferase SET domain containing 2 (SETD2) [116]. The stoichiometry of Lys40 methylation and acetylation within MTs is not known, but a recent study in mouse cortical neurons showed, as expected, an inverse relationship between the levels of MT Lys40 trimethylation (which decreases between embryonic day 17.5 and adulthood) and Lys40 acetylation (which increases during this period) [155]. Moreover, another study showed that α-tubulin Lys40 trimethylation is able to rescue the defects of radial migration and morphological transition of cortical neurons caused by α-tubulin Lys40 acetylation deficiency [156]. Another crosstalk may exist between tubulin tyrosination and α-tubulin Lys40 acetylation. In fact, a correlation between tubulin acetylation and detyrosination was found in α-TAT1 that is acutely depleted and knock-out murine embryonic fibroblasts, in which both acetylated and detyrosinated MTs levels appeared to be decreased [157]. More recently, it was found in primary neurons that tubulin re-tyrosination can control acetylated tubulin levels and that tubulin acetylation is affected by the tubulin tyrosination/detyrosination cycle [158]. Interestingly, a crosstalk between the tubulin tyrosination/detyrosination cycle and neuron-specific Δ2 tubulin also occurs. The pool of detyrosinated tubulin can be further acted upon by cytosolic carboxy peptidases (CCPs), which catalyze the removal of the terminal glutamate residue from tubulin, converting detyrosinated tubulin into Δ2 tubulin [159]. Given that the enzyme catalyzing tubulin tyrosination, tubulin tyrosine ligase, is unable to use Δ2 tubulin as substrate, the formation of Δ2 tubulin is irreversible and, besides removing it from the tubulin tyrosination/detyrosination cycle [150], may also affect the levels of α-tubulin acetylation.

Since formation/removal of tubulin modifications can occur in free tubulin heterodimers or MTs, we may wonder if tubulin co-factors play a regulatory role in remodeling tubulin PTMs patterns in MTs in response to specific cues by regulating tubulin recycling, degradation, and quality control. Undoubtedly, the set of enzymes that catalyze and revert tubulin PTMs and their patterns and mechanisms of regulation are another complex layer in the regulation of MT organization, function, and dynamics, with profound importance for cell homeostasis. Thus, it is expected that abnormal tubulin posttranslational modifications may contribute to various human diseases, such as cilia, neuronal, muscle, blood disorders, and cancer (reviewed here and in [160]).

The combination of tubulin isotypes and isoforms patterns contributes to modulating/changing/fine-tuning intrinsic features of MTs, such as stability and dynamics, mechanical properties (flexibility and resistance to mechanical stress), polymerization/depolymerization rates, and the ability to form specific MTs organizations and structures. Additionally, these patterns probably affect the interactions of MTs with motor proteins and MAPs, which will also have consequences on MTs’ intrinsic properties. The generated diversity will allow cells to cope with different environments, signals and, in the case of metazoans, with different tissue architectures and physiology challenges. Supporting these ideas is the fact that distinct patterns of tubulin isotype expression and tubulin PTMs can be observed in different cell types during the cell cycle and development [161]. Tubulin PTMs may occur and be predominant in specific sets of MTs that coexist inside the same cell. Therefore, specific tubulin PTMs profiles can be found in MTs of centrioles, cilia/flagella, kinetochore fibers, midbody, and axonal and cone MTs (for review [161]), or even between adjacent MTs, as in the case of the A and B tubules of axonemal doublets. In flagella/cilia of *Chlamydomonas* and *Tetrahymena*, the B tubule is highly glutamylated [162,163], whereas, in algae A, the tubule is enriched by detyrosinated tubulin [164].

## 2. The Tubulin Acetyltransferases and Deacetylases

### 2.1. Tubulin Acetyltransferases

The acetylation/deacetylation of tubulin is mediated by the action of acetyltransferases and deacetylases. Despite α-tubulin Lys40 being the most well studied and possibly the predominant acetylation site in MTs, mass spectrometry studies have identified other potential acetylation sites in both α- and β-tubulins. Two of the residues that are consistently identified in these studies are the lysine residue 394 (Lys394) of α-tubulin and lysine residue 252 (Lys252) of β-tubulin [134,135,136,137,138,139].

αTAT1 is the enzyme responsible for catalyzing nearly all α-tubulin Lys40 acetylation [145,165], as shown by studies with transgenic mice, where deletion of the gene coding for the enzyme leads to nearly complete loss of tubulin acetylation [166,167,168]. αTAT1 belongs to the Gcn5-related N-acetyltransferase (GNAT) superfamily, presenting a catalytic domain homologous to histone acetyltransferases [169,170,171,172,173]. The enzymes in the GNAT family use acetyl-coenzyme A (acetyl-CoA) as the donor of the acetyl group that will be transferred to a primary amine—the ε-amino group of Lys40, for α-tubulin [174].

However, studies performed in *Drosophila* showed that overexpression of αTAT1 does not affect Lys394 acetylation levels; therefore, this acetylation is likely catalyzed by a different, yet unknown, acetyltransferase [175]. In β-tubulin, the acetylation of Lys252 is also not dependent on αTAT1, but on San acetyltransferase [176]. In fact, αTAT1 appears to be unable to catalyze the acetylation of other lysine residues at tubulin MTs besides Lys40, probably due to a very specific active site [145,177]. Nevertheless, in animal cells, other enzymes colocalize with MTs and they are also able to alter the levels of α-tubulin acetylated Lys40, such as ARD1-NAT1 [178], GCN5 [179], elongator protein 3 [180], and N-acetyltransferase 10 [181]. While αTAT1 is the major α-tubulin acetyltransferase, these other acetyltransferases appear to be responsible for minor α-tubulin Lys40 acetylation events: for example, ARD1-NAT1 catalyzes MT acetylation in dendrites; and, elongator protein 3 regulates α-tubulin in order to promote the migration and differentiation of cortical neurons; and, N-acetyltransferase 10 concentrates at the midbody, where it regulates MT acetylation during cytokinesis [178,180,181]. It is important to mention that San acetyltransferase has an important role in sister chromatid cohesion in *Drosophila* and human cells [182,183]. As mentioned, San acetyltransferase was also discovered to be an acetyltransferase, catalyzing the acetylation of Lys252 in free β-tubulin. This modification affects the assembly of the α/β-tubulin heterodimer and, consequently, MT polymerization and dynamics. In fact, San depletion in human HeLa cells increases the regrowth rate of MTs after nocodazole treatment [176].

Several organisms rely on αTAT1 activity to catalyze the acetylation of α-tubulin. In *Tetrahymena*, *C. elegans*, zebrafish embryos, and human HeLa cells, αTAT1 deficiency leads to the loss of Lys40 acetylation [165]. αTAT1 depletion in *Tetrahymena* alters the sensitivity to tubulin-targeting compounds [165,184], and in *Toxoplasma*, the cells present an altered morphology and are unable to undergo mitosis [185]. In human RPE-1 cells, the depletion of αTAT1 leads to a high frequency of MT depolymerization events, while overexpressing the enzyme increases the number of nocodazole-resistant MTs. However, the number of nocodazole-resistant MTs can be restored in the absence of αTAT1 by removing compressive forces that are applied to the cell [157]. The migration of cancer cells is affected by αTAT1, as well [186]. Interestingly, in mice fibroblasts, the absence of αTAT1 increases MT resistance to nocodazole, while αTAT1 overexpression destabilizes the MTs and increases their dynamics. Although these results oppose those observed in human cell lines, the authors further observed that the overexpression of catalytically inactive αTAT1 destabilizes the MTs, even if the acetylation levels were unaffected. Therefore, overexpressing catalytically active or inactive αTAT1 affects MT dynamics, suggesting that this destabilizing effect may occur due to a regulation mechanism other than MT acetylation [167,187]. Remarkably, αTAT1 is important in cells for functions other than the acetylation of α-tubulin. In *C. elegans*, αTAT1 is expressed solely in mechanosensory neurons [105,145]. The absence of αTAT1 impairs touch sensation [145,165,188,189], a phenotype that can be reversed by catalytically inactive αTAT1 [190]. Although α-tubulin is the major substrate for αTAT1, the enzyme can also catalyze the acetylation of other substrates, such as cortactin [186], an actin-binding protein with an important role in cell migration and adhesion [191]. Additionally, αTAT1 is able to catalyze its self-acetylation in a process that directly impacts α-tubulin acetylation [187]. αTAT1 is also enriched in structures, such as focal adhesions and clathrin-coated pits, where it can catalyze the acetylation of α-tubulin due to its interaction with the clathrin-assembly protein AP2 [192]. These alternative αTAT1 substrates may help explain αTAT1 phenotypes that seem independent of α-tubulin acetylation.

αTAT1 has a substrate preference towards polymerized MTs when compared to free α-tubulin [145]. As α-tubulin Lys40 is localized in the MT lumen, αTAT1 must enter the MTs to acetylate this amino acid residue [193,194]. There are two main hypotheses for how the enzyme can enter the lumen of MTs: either αTAT1 enters through the open MT ends and diffuses along the length of the MTs, or the enzyme permeates through irregularities in the MT wall. Several observations support the first hypothesis. Firstly, the ciliary axoneme is acetylated, in vitro, from the ends, with αTAT1 diffusing through the MT lumen [165].

Additionally, Szyk and colleagues (2014) [195] used immunofluorescence experiments to show that αTAT1 colocalizes with the MTs, with a higher affinity for MT ends, possibly because of the high density of exposed luminal sites [195,196,197]. However, these experiments do not allow us to distinguish if the enzyme is localized in the MT lumen or on the external surface of the MT. Additionally, the presence of discontinuous acetylation patterns cannot be explained [195]. Furthermore, a mathematical model suggested that diffusion of αTAT1 in the MT lumen would happen slowly, which could be explained by the enzyme’s frequent rebinding [196,198]. Consequently, it was observed that long-lived MTs are those with higher levels of acetylation across their entire length, as they are those that last long enough for αTAT1 to diffuse and acetylate them [195,197]. Besides the slow diffusion rate, the diffusion hypothesis is also challenged by the fact that, in living cells, MTs are highly dynamic structures that bind several tip-binding proteins [199], leading to high occupancy of their extremities and consequently obstruct the entry of αTAT1 in the lumen of MTs. Concerning the second hypothesis, it is assumed that αTAT1 can permeate the MT through defects in the MT wall. This would require a high number of irregularities on the surface of the MT for the enzyme to enter the MT and acetylate its entire length. The defects would, therefore, be transient, providing αTAT1 with frequent entry points into the MT lumen [196,200,201]. This is also supported by αTAT1 binding to the external wall of MTs, allowing the enzyme to search for those access sites [197,202]. It was previously mentioned that Szyk and colleagues (2014) observed discontinuous patterns of acetylation, which the diffusion of the enzyme could not justify [195]. However, these patterns could be explained by transient defects in the MTs lattice, which would allow αTAT1 to enter the MT lumen and catalyze Lys40 acetylation discontinuously [195]. Altogether, the enzyme is apparently able to access the MT lumen through both the extremities of the MTs and the defects in their walls, but a quantitative analysis of the contribution of each putative entry for αTAT1 is missing [203].

### 2.2. Tubulin Deacetylases

So far, we have examined the main enzyme responsible for α-tubulin acetylation. However, in what concerns the deacetylation of this protein, there are two important players: histone deacetylase 6 (HDAC6) [204,205] and SIRTUIN 2 (SIRT2) [206]. These enzymes belong to each of the two families of lysine deacetylases (KDACs), also known as histone deacetylases (HDACs) [207] and have a role in epigenetic gene expression silencing.

SIRT2 is one of seven members of the Sirtuin family (also known as class III KDACs), which encompass NAD^+^-dependent lysine deacetylases and ADP-ribosyltransferases [208]. SIRT2 is the only member of this family located predominantly in the cytoplasm, where it catalyzes the deacetylation of α-tubulin [206]. However, the enzyme can transiently shuttle to the nucleus during the G2/M transition and catalyze the deacetylation of histone H4, therefore modulating the condensation of chromatin during metaphase [209,210,211]. Additionally, SIRT2 overexpression increases the length of mitosis, suggesting an important role for the enzyme in the regulation of mitosis [210]. This is further supported by the identification of other SIRT2 substrates, such as CDH1/CDC20 [212] and CDK9 [213]. While CDH1/CDC20 is important in the transition from metaphase to anaphase, CDK9 has a crucial role in maintaining genome integrity. On the other hand, SIRT2 itself is regulated by cell cycle-dependent kinases (Cdk1, Cdk2, and Cdk4) [214]. Consequently, the mutual interaction between SIRT2 and these proteins illustrates the importance of this enzyme in the cell cycle regulation and establishes a link between SIRT2 and cancer, a disease characterized by genomic instability and aberrant mitosis [212,213]. It is also important to mention that SIRT2 has been associated with neurotoxicity and neurodegenerative diseases, as it influences the aggregation of α-synuclein, huntingtin, amyloid-β peptide, and Tau protein [215,216,217,218].

HDAC6 is mainly a cytoplasmic deacetylase, which can be shuttled to the nucleus by interacting with the nuclear import protein importin-α [219]. In addition to α-tubulin, this enzyme has several well known substrates, such as heat shock protein 90, cortactin, peroxiredoxins I and II, heat shock transcription factor-1, and the E3 ubiquitin ligase TRIM50 (tripartite motif-containing protein 50) [220,221]. HDAC6 regulates the degradation of misfolded/aggregated proteins [222,223] and the mitochondrial transport in hippocampal neurons [224], assuming an important role in neurodegenerative diseases [225]. Similarly to SIRT2, HDAC6 is also associated with tumorigenesis and metastasis, and the inhibition of this enzyme is an interesting approach to treating several types of cancer [226,227]. HDAC6 also influences the immune response [205]. This enzyme has a ubiquitin-binding zinc-finger in its C-terminal region [228], and it binds ubiquitin, delaying the recognition of ubiquitinated proteins by the proteasome [223,229].

Initially, it was thought that α-tubulin deacetylation occurred in free α/β-tubulin heterodimers, since deacetylation correlates with MT depolymerization [230]. Since then, studies have shown that HDAC6 can catalyze the deacetylation of polymerized MTs in vitro [204,231]. Nevertheless, more recent experiments highlighted a substrate preference towards free tubulin heterodimers [232,233]. In the case of SIRT2, it can act on both soluble tubulin and polymerized MTs [206]. The two enzymes co-localize with MTs, as well as with each other. Despite this interaction, HDAC6 and SIRT2 are able to individually catalyze the deacetylation of α-tubulin, in vitro and in vivo [148,204,206,231]. Considering that HDAC6 catalyzes the deacetylation of MTs, it needs to access the MT lumen, similarly to αTAT1. As HDAC6 interacts with EB1, a protein that localizes at the MT plus end, it has been suggested that the enzyme could permeate the MT lumen through this interaction [234].

Several studies on the impact of the overexpression/depletion of the deacetylation enzymes on cells have been made. Deletion of *sirt2* in mice does not change the levels of α-tubulin acetylated Lys40 in the brain [235]. On the contrary, the deletion of the murine hdac6 gene leads to α-tubulin hyperacetylation [205], which has led to the suggestion that HDAC6 is the major tubulin deacetylase in vivo. However, it is possible that the two enzymes catalyze the deacetylation of different subsets of MTs. In fact, Skoge and Ziegler [236] found that HDAC6 inhibition led to a general hyperacetylation of the MT network throughout the cell, whereas hyperacetylation induced by SIRT2 inactivation was limited to perinuclear MTs. Following HDAC6 overexpression, hyperacetylation of these perinuclear MTs was maintained, while reactivation of SIRT2 restored the basal acetylation level and a normal MT network. Moreover, siRNA-mediated depletion of either SIRT2 or HDAC6 increased the levels of acetylated α-tubulin, with HDAC6-silencing being more effective. In mammal cells, HDAC6 knockdown stabilizes the MTs, as the increase in Lys40 acetylation leads to nocodazole-resistant MTs [231,237]. HDAC6-depleted mice are viable and fertile, but they present some phenotypes. Firstly, their fibroblasts are enriched in stable MTs, with shorter depolymerization events [237]. Regarding motility, the overexpression of HDAC6 leads to chemotactic cell movement, with fibroblasts moving about 3.5-fold faster than wild-type cells [204,238]. However, it is unclear whether HDAC6 affects motility through increased MT acetylation levels in the leading edge of the fibroblast or through deacetylation of the actin cytoskeleton (which also affects cell motility) [239]. It was also observed that HDAC6 depletion could cause alterations in cells independently from its catalytic activity. Zilberman and colleagues (2009) observed that inhibiting HDAC6 leads not only to an increase in MT acetylation levels, but also to a decrease in MT dynamics [234]. On the contrary, HDAC6 depletion had no effect on MT dynamics, which suggested that the binding of HDAC6 to the growing extremity of the MT could physically affect MT polymerization/depolymerization independently of its deacetylase activity [234].

Ciliogenesis is also affected by the action of both HDAC6 and SIRT2, as the enzymes promote cilia disassembly in mammalian cells through the deacetylation of α-tubulin. While primary cilia are mainly lost in overexpression conditions, HDAC6 or SIRT2 depletion increases the number and length of cilia [240,241,242,243,244]. Accordingly, in RPE-1 cells, the inhibition of HDAC6 dependent axoneme’s deacetylation blocks the resorption of primary cilia [240]. The effect of HDAC6 in cilia may also occur through the actin cytoskeleton, as well as the enzyme deacetylates cortactin. Actin polymerization then occurs around the base of the cilium, and the change in actin cytoskeleton dynamics will contribute to shortening the cilia length [244,245].

In vivo, tubulin acetylation levels depend not only on the activity of acetylases/deacetylases, but they also depend on factors that regulate their activities. One example is the acetyltransferase p300, an enzyme that downregulates the expression of αTAT1 and the activity of HDAC6 and SIRT2 [143,246,247]. The availability of acetyl-CoA, the donor of the acetyl group for the acetylation reaction, as well as NAD^+^, also regulates MT acetylation levels. The synthesis of cytosolic acetyl-CoA from citrate and acetate is catalyzed by ATP-citrate lyase and by acyl-CoA synthetase short-chain family member 2, respectively [248]. When the activity of these enzymes is reduced, the acetylation of MTs will also be impaired, as there is less available acetyl-CoA and, therefore, less acetyl groups that can be transferred onto Lys40 residues [248]. The levels and subcellular localization of acetyl-CoA are also an important indicator of the metabolic state of the cells: when there is a high need to produce energy, acetyl-CoA is formed from pyruvate in mitochondria and oxidized in order to promote ATP synthesis; when the cell has excess energy, acetyl-CoA can be exported from mitochondria to the cytosol as citrate and resynthesized from citrate, becoming abundant in the nucleus and cytoplasm, where it may be used for lipid biosynthesis and histone/protein acetylation.

On the other hand, MT deacetylation via SIRT2 is NAD^+^-dependent [206]. During this reaction, NAD^+^ acts as a co-substrate that is converted into nicotinamide, while the acetyl group is removed from the MTs to originate O-acetyl-ADP-ribose [249]. This co-substrate will, therefore, influence MT acetylation levels. Additionally, NAD^+^ has an important role in energy production in the cells: in glycolysis and in the tricarboxylic acid (TCA) cycle, NAD^+^ is reduced to NADH; NADH is then used as an electron donor to the electron transport chain in the mitochondria for ATP production through oxidative phosphorylation [250,251]. Interestingly, both NAD^+^ and acetyl-CoA are relevant molecules for the energetic state of the cells and for the acetylation levels of MTs. This suggests a link between MT acetylation and the energetic state of the cells. Therefore, the acetylation levels of proteins, such as α-tubulin, may reflect the metabolic and energetic state of the cells [252].

## 3. Tubulin Acetylation: Structural and Functional Implications

Tubulin acetylation has been one of the most puzzling tubulin PTMs studied throughout the years. To this day, although several breakthroughs have been made, the role of tubulin acetylation is far from being completely understood.

### 3.1. Structural Implications of Tubulin Acetylation

Since the early studies on tubulin acetylation at Lys40, scientists have found an association between this PTM and stable MTs [230,253]. Several in vitro studies showed that acetylated MTs are more resistant to depolymerization when exposed to cold and when treated with depolymerizing drugs (nocodazole or colchicine) [230,254]. However, whether this MT stabilization was due to the acetylation or if tubulin acetylation occurred because the MTs were long-lived, as well as how this stabilization was achieved, remained open questions [231,255,256,257,258]. For many years, the data obtained from several in vivo studies did not allow any conclusion, since the results supported different roles for tubulin acetylation. While some studies showed that tubulin acetylation resulted in more stable MTs [231,237], others showed no or even a destabilizing effect [257,259].

The molecular structure of acetylated and deacetylated tubulin is highly similar [202], so how the acetylation could contribute to the previously described alterations in MT stability remained unclear. In 2012, Cueva et al., using molecular dynamics simulations, proposed that Lys40 acetylation could be involved in establishing salt bridges between adjacent α-tubulins, leading to a rearrangement in the inter-protofilament angle [260]. This suggested that α-tubulin Lys40 acetylation could contribute to the mechanical properties of the MTs. Microtubules bend in response to various cellular cues and stresses, which requires adjacent protofilaments to slide in relation to one another. This process depends on the interactions between tubulin molecules of adjacent protofilaments [261,262]. These ideas have been supported by recent studies that have given more information about how tubulin acetylation could contribute to changes in the mechanical properties of MTs. One structural study indicates that the acetylation of Lys40, which is located in an unstructured loop of α tubulin, reduces interactions between protofilaments, potentially promoting protofilament sliding and enhancing MT flexibility [263]. As a result, acetylation of Lys40 alters the mechanical properties of MTs in cells. This new research suggests that acetylation of α-tubulin at Lys40 helps to prevent MTs from mechanical aging, in which they lose their rigidity from repetitive bending [264], stopping MT breakage and extending the MTs’ lifespans within cells [157].

Interestingly, the changes in the flexibility of MTs do not seem to be the only structural alteration promoted by tubulin acetylation. In a *C. elegans* ortholog of αTAT1 (MEC-17) mutant, the MTs of touch receptor neurons display a variable number of protofilaments, even when the mutant is rescued with an inactive MEC-17 [190,260,265]. Thus, the acetylation activity of MEC-17 seems essential to control the protofilament number in these neurons. This variation in protofilaments number could also be explained by the model where tubulin acetylation regulates the lateral interactions of tubulin protofilaments [260,263].

Beyond its role in mechanical stabilization, the question of whether Lys40 tubulin acetylation is involved in regulating MT dynamics was posed right from the beginning. Earlier work, using tubulin purified from calf brain, suggested that acetylation of α-tubulin at the Lys40 residue did not affect the MTs dynamics in vitro [256]. However, the experimental design of these early experiments had some flaws. Tubulin in neurons is heavily acetylated [266]; therefore, the tubulin used as a control in those experiments would already have high acetylation levels. More recent studies have shed some light on this issue. In 2017, Portran et al. [264] showed that the self-assembly rate of acetylated tubulin was much slower than that of deacetylated tubulin. Interestingly, no differences were found in polymerization rates for acetylated and deacetylated tubulin when the measurements were made with the MTs already formed. However, in this scenario, acetylated MTs had a disassembly rate threefold faster than deacetylated MTs [264]. The authors suggested, as they have for the mechanical stability against breakage, that this is consistent with a model where tubulin acetylation at Lys40 modulates lateral, but not longitudinal, interactions in the protofilaments [264].

Another way tubulin acetylation could control MT stability is by regulating MT severing proteins. Several studies have shown that the MT-severing protein katanin preferentially severs acetylated MTs [267,268]. Why katanin severs these more stable MTs has yet to be fully understood, but this process may be related to the facilitation of the transport of short MTs around the cell and not to their depolymerization. In fact, the transportation of stable acetylated MTs would prevent their depolymerization during the process [268]. Similar to katanin, fidgetin also shows a preference for specific groups of MTs. However, unlike katanin, the preferred group of MTs varies in different organisms, since, in mouse neurons, fidgetin presents a higher affinity to sever deacetylated MTs, while in *Drosophila*, it shows a preference for acetylated MTs [269].

### 3.2. Functional Impact of Tubulin Acetylation

The cell’s three major groups of MT structures can present α-tubulin acetylation on Lys40: cilia, centriole, and the MT cytoplasmic network (Figure 2) [230,270]. Although the general consequences of the acetylation in the MTs are common to the different groups, some particularities are worth dissecting in each of these groups.

#### 3.2.1. Cilia Microtubules

Tubulin acetylation at Lys40 was first discovered in the cilia of *Chlamydomonas* [254,271]. Since then, the study of α-tubulin acetylation has been intensively associated with cilia. α-tubulin Lys40 acetylation is the most abundant acetylation site in cilia MTs [165], with this PTM being present in most axonemal tubulin, both in the central pair and in the outer MTs [184,253]. The acetylation of axonemal tubulin seems evenly distributed, whereas other PTMs seem to accumulate closer to the proximal part of the cilia [108].

The association of acetylated tubulin to ciliary MTs seems logical, since these are stable MTs. Besides that, αTAT1 localizes in motile and primary cilia of different types of cells [272]. However, the specific role of this PTM in the cilia structure and function remains far from being completely understood. Some studies have shown that tubulin acetylation is critical for cilia assembly and stability. Shida and coworkers (2010) showed that tubulin acetylation is required for the correct assembly of cilia in mammalian cells [145]. Additionally, lithium treatment in human fibroblasts induced cilia elongation through tubulin acetylation [273]. Mutant mice for αTAT1 display subfertility phenotypes, with the authors suggesting that acetylation of the axonemal tubulin is crucial for sperm motility [167]. As mentioned, in human hTERT-RPE1 cells, HDAC6 leads to cilia disassembly, which may result from a combination of deacetylation of α-tubulin with that of cortactin [244].

Several ciliopathies and other diseases, such as Bardet-Biedl syndrome or Huntington’s disease, present altered tubulin acetylation, causing cilia to malfunction [274,275]. On the contrary, many other studies show that tubulin acetylation is unnecessary for cilia assembly. For example, Kalebic and colleagues (2013) showed that, although mutant mice for αTAT1 had defects in the functioning of sperm flagella, cilia in other animal cells were present and only showed mild defects, even in the absence of tubulin acetylation [167]. In the ciliate protozoa *Tetrahymena* and the algae *Chlamydomonas*, mutants for αTAT1 or expressing a Lys40Arg non-acetylatable α-tubulin have relatively normal cilia [165,184,276].

Tubulin acetylation may also have a role in controlling ciliary motor proteins. In fact, in vitro, Lys40 acetylation seems to increase the rate of motility of dynein from the axoneme outer arm [277]. The observation helps to explain why mice lacking αTAT1 show defects in the functioning of sperm flagella, resulting in reduced fertility [167]. In the case of kinesin-1, results appear to be more conflicting. Initial studies have shown that α-tubulin Lys40 acetylation increases the speed of kinesin-1 motility in vitro [278]. However, more recent studies observed that this PTM might not influence the motility of kinesin-1 in axonemes [279,280]. Interestingly, MIPs containing the DM10 domain (the axonemal FAP67 and RIB72) were found to bind to the αLys40 loops [95]. However, if these MIPs are recruited to this localization by α-tubulin Lys40 acetylation, or if the binding of these proteins prevent the α-tubulin Lys40 acetylation, requires further investigation.

#### 3.2.2. Centrioles

Microtubules in mature centrioles are heavily and entirely acetylated [281]. However, the precise role of acetylation in centrioles is far from being understood. Still, based on the current knowledge about the impact of α-tubulin Lys40 acetylation in MTs, i.e., allowing them to bear more mechanical stress and rendering them more flexible [157,263,264], we can start to envisage how helpful this PTM can be in centrioles. Centrioles are relatively stable structures that must endure complete cell cycles without disassembling [281]. Besides that, due to their function in the cell and its structure, centrioles are subjected to a series of mechanical stresses, especially torsion, due to their architecture and functions [282]. Therefore, it is tempting to suggest that centrioles’ MTs have high levels of acetylation, so they can be flexible enough to deal with the mechanical challenges posed by their functions [282]. The timing of centriole MT acetylation during their maturation is also interesting. Procentrioles start to show acetylation of their MTs during the early steps of formation, but this acetylation shows a slight delay concerning MT growth. By the time the centriole matures, the entire MT wall will be acetylated [283].

#### 3.2.3. Cytoplasmic Microtubules Arrays

Cytoplasmic MT acetylation is much more heterogeneous than that of cilia and centrioles. Although α-tubulin Lys40 acetylation is present in most cells, its abundance and distribution pattern varies according to cell type, cell life cycle, and cell region [284]. Among the several cell types, neurons have a high level of α-tubulin Lys40 acetylation, with a higher density of this PTM in the axons, while dendrites are globally less acetylated [253,285,286]. In fact, studies have shown that MTs display a gradient of acetylation toward the end of the axon [285,287]. Surprisingly, although neuronal tubulin is highly acetylated, mutant mice for the αTAT1 gene have a low neurological impairment [166]. The major defects observed at the neurological level in these mutant mice are related to the loss of touch sensation [288]. Noteworthy, similar mutants in other organisms (e.g., *Drosophila* and *C. elegans*) display the same type of phenotype [190,289,290].

More recent studies in neuronal tubulin acetylation have shown the existence of two neuronal MT populations [291]. In dendrites, MTs have a heavily acetylated population of MT organized in bundles and another population with very low acetylation levels [291]. These two populations of MTs presented different polarities, with the heavily acetylated MTs being associated with the retrograde transport driven by kinesin-1 and the low acetylated MTs being associated with the anterograde transport driven by kinesin-3 [291]. In fact, several other studies have suggested a relationship between MT acetylation status and the binding of molecular motors dynein and kinesin, especially in neurons [292,293,294,295]. Mitochondrial movements around the cell, probably driven by molecular motors associated with MTs, have been shown to occur favorably using acetylated MTs [296,297]. The same phenomenon is observable in vesicular trafficking, where vesicles (that can even contain αTAT1) are transported along acetylated MTs [298].

Despite this evidence for the role of tubulin acetylation in regulating intracellular trafficking, a complete picture is far from being attained. As mentioned, in vitro assays showed no influence of tubulin acetylation in kinesin-1 movement along MTs [279,280]. Additionally, if tubulin acetylation severely affected axon trafficking, it would be expected that organisms with altered tubulin acetylation levels would have severe neurological defects, but that is not what was found in several studies, as explained before [166,205,288]. This apparent lack of consistency in the results opens the door to other factors that can contribute to this process, together with tubulin acetylation [299]. There are several pieces of evidence that the combination of multiple PTMs [293,300], the existence of adapters and helpers for motor proteins [301,302], and the existence of MAPs that can bind to MTs and create blocks for motor proteins movement [303,304] are all factors that can modulate the effect of tubulin acetylation in intracellular trafficking, and they should be explored in the following years [299].

In fact, the crosstalk between MAPs and the Lys40 α-tubulin acetylation is far from being completely understood. Several studies have shown a relationship between distinct MAPs and the Lys40 α-tubulin acetylation [305,306,307,308,309,310]. Tau protein has been one of the most intensively studied MAPs. Early studies have shown that Tau overexpression increases MT Lys40 α-tubulin acetylation levels and corresponding stabilization against MT depolymerizing agents [305,306]. The mechanism by which this increase in Lys40 α-tubulin acetylation is achieved seems to be related to the binding of Tau protein to HDAC6, causing a decrease in the activity of this enzyme [308]. Besides affecting Lys40 α-tubulin acetylation levels, Tau protein seems also to bind preferentially to acetylated and tyrosinated MTs [307]. Interestingly, Lys40 α-tubulin acetylation levels also seem to affect Tau protein functions. In 2017, Mao and colleagues showed that cells present an acetylation-mimicking mutation in α-tubulin rescued the Tau-induced MT defects [309].

Besides Tau, other MAPs have been associated with Lys40 α-tubulin acetylation. This is the case for MAP1B and MAP2c, which have been shown to increase Lys40 α-tubulin acetylation levels when overexpressed [305]. On the other hand, the MAP α-synuclein has recently been shown to decrease the Lys40 α-tubulin acetylation levels when overexpressed, as well as expressing a reduced binding to deacetylated MTs [310].

Another process where cytoplasmic MT acetylation seems to be involved is cell division. Acetylation of α-tubulin Lys40 is highly abundant in the mitotic spindle, midbody, and kinetochore MTs [311,312]. A general deacetylation of the MTs occurs during telophase, except for the midbody [313,314,315]. Although the spindle MTs are enriched in tubulin MTs, α-tubulin Lys40 acetylation does not seem to affect the polar chromosome congression [311]. Interestingly, the enzymes involved in MT acetylation/deacetylation (αTAT1, HDAC6, and SIRT2) have a regulation dependent on the cell cycle stage and seem to affect cell cycle progression [270,316,317,318,319]. However, as stated previously, mice lacking functional αTAT1 and, consequently, showing no acetylation, exhibit relatively normal cell division and proliferation [166,167], although their cultured cells have reduced levels of contact inhibition and a low number of focal adhesions during proliferation [168]. The same seems true for HDAC6 and SIRT2 mutant mice, which showed no severe defects in cell proliferation [205,235]. As already mentioned for intracellular trafficking, these apparently contradictory results seem to suggest the existence of other mechanisms involved in the regulation of cell cycle and cell division, beyond tubulin acetylation, that can compensate for its effects.

Cytoplasmic tubulin acetylation also seems to regulate cell shape and migration, although this last hypothesis remains controversial. One of the most striking examples of the participation of tubulin acetylation in cell structure is the marginal band of blood platelets formed by a bundle of heavily acetylated MTs that maintain the discoidal shape of resting platelets [320,321]. Upon platelet activation, the MTs suffer an abrupt deacetylation catalyzed by HDAC6, leading to their reorganization and allowing a change in platelet shape [321]. For cell migration to occur, an intracellular rearrangement must happen, with several organelles being re-localized. Evidence shows that tubulin acetylation is involved in this process by contributing to the organelles’ reorganization [322] or reorganizing MTs near the cell’s leading edge [192]. Loss of αTAT1 and overexpression or inhibition of HDAC6 also seems to affect cell migration, with cells presenting an increased chemotactic movement when tubulin was deacetylated [186,204,259,323,324]. Cell adhesion to the substrate, a process crucial for cell migration, has been shown to be affected by changes in the level of tubulin acetylation. Although there are some contradictory results, loss of either HDAC6 or αTAT1 has been associated with an increase in the number of focal adhesions [237,325]. This opens the possibility of tubulin acetylation playing a crucial role in tissue morphogenesis. In fact, recent studies have shown that, during tissue morphogenesis, acetylation of MTs is critical for the penetrative capacity of cells undergoing radial intercalation in epithelia [326]. Additionally, Seetharaman and colleagues have shown that the protein Talin recruits αTAT1 to the focal adhesions where the MT acetylation is critical for fine-tuning the mechanosensitive cell adhesion and migration [327]. This role of acetylated α-tubulin Lys40 in cell migration has been explored in oncology, with several studies targeting MT acetylation as a chemotherapeutic approach [328].

### 3.3. Tubulin Acetylation beyond Lys40

Although most studies on tubulin acetylation have been focused on acetylation at the Lys40 residue, several studies have shown that both α- and β-tubulins can also be acetylated in other lysine residues [135,136,139]. Still, acetyl incorporation studies have shown that α-tubulin is far more acetylated than β-tubulin [329]. Although there is not yet extensive work on the function of each of these other tubulin acetylation sites, some data are already available. The β-tubulin Lys252 residue, which can be acetylated, localizes at the interface between the two tubulins in the heterodimer, and its acetylation seems to affect the α/β tubulin interaction and, consequently, the stabilization of the heterodimer [176]. In fact, acetylation of this β-tubulin residue seems to impact MT polymerization directly. On the other hand, the knockdown of San acetyltransferase, which catalyzes the acetylation of β-tubulin Lys252, resulted in faster MT regrowth after cold-shock depolymerization, and the MT depolymerization rate was not affected [176]. Acetylation of other α-tubulin lysine residues besides Lys40 has also been studied and shown to have some impact on MT polymerization/depolymerization rates [136]. Of those, Lys394 acetylation has been shown to be essential for MT stability and to be regulated by HDAC6 deacetylase [175]. This lysine residue locates at the interface of the α/β-tubulin heterodimer in the MT surface, a region that suffers a conformational change as the dimers are added to the MT [330]. Studies overexpressing a Lys394Arg mutant α-tubulin, which cannot be acetylated, showed poor tubulin incorporation during MT assembly [136].

With the discovery and increasing data about these new tubulin acetylation sites, the question arises of whether these acetylation sites influence each other in controlling MT properties [299]. However, we are still far from understanding how these PTMs can collectively contribute to regulating MT function, and many more studies on the role of these new acetylation sites are needed (Table 1).

## 4. Tubulin Acetylation and Stress Conditions

Increased levels of MT acetylation (hyperacetylation) at α-tubulin Lys40 have been found in cells exposed to several cellular stresses and lead to increased cell survival [227,246,331]. MT hyperacetylation has been observed in human (HeLa, RPE-1) and mouse (embryonic fibroblast, 3T3) cell lines under nutrition starvation [246,331], high glucose [227], salt stress by excess NaCl [246], oxidative stress caused by high levels of hydrogen peroxide (H_2_O_2_), and after exposure to several chemical agents, e.g., taxol [230], Ni^2+^ [333], the synthetic hormone ethinyl estradiol, the insecticide methoxychlore, the apoptotic agent staurosporine [227,246], and physical agents, such as UVC radiation [334]. Despite being observed in several cell lines, stress-dependent MT hyperacetylation does not seem to be a general cell response to stress, since, in pulmonary vascular endothelial cells, both LPS-induced and H_2_O_2_-induced oxidative stress decreased MT tubulin acetylation [335]. Therefore, what seems to be a general cell response to stress is the occurrence of changes in the levels of MT acetylation.

### 4.1. Mechanisms of Microtubules α-Tubulin Acetylation Regulation under Stress

Stress-dependent tubulin hyperacetylation is not due to decreased activities of deacetylases, since deacetylase activity remains unchanged in lysates from stressed cells when compared to control cells [246]. However, αTAT1 participates in tubulin hyperacetylation in response to stresses, since, in cells where αTAT1 was knocked down using siRNA, or in mutant Atat1^−/−^ mouse fibroblasts, stress conditions failed to induce tubulin acetylation [227,246].

H_2_O_2_ is a possible mediator of the signaling processes, leading to tubulin hyperacetylation under several stresses, since N-acetylcysteine decreases the levels of MT acetylation induced not just by H_2_O_2_, but also by ethinyl estradiol and NaCl [246]. This reduced α-tubulin acetylation in the presence of N-acetylcysteine has led to the proposal that stress-dependent tubulin hyperacetylation involves activation of AMP-activated protein kinase (AMPK) by increased levels of H_2_O_2_, originating in the mitochondria, leading to increased αTAT1 activity [246]. AMPK, a sensor of the energetic state of the cell [336], is a regulator of acetyl-CoA homeostasis. AMPK catalyzes the phosphorylation and inhibition of acetyl-CoA carboxylase, which catalyzes the carboxylation of acetyl-CoA to malonyl-CoA, the initial reaction in fatty acid synthesis. As already referred, since acetyl-CoA is a substrate for αTAT1, AMPK activation could lead to increased αTAT1 activity by increasing the cellular level of acetyl-CoA [337,338,339].

AMPK activation involves phosphorylation of the AMPKα subunit, which is dependent on upstream AMPK kinases, identified as liver kinase B1 (LKB1), Ca^2+^/calmodulin-dependent protein kinase kinase 2 (CaMKK2), and transforming growth factor-β-activating kinase 1 (TAK1) [340]. H_2_O_2_ is known to activate AMPK, either directly through oxidation of the AMPKα subunit [341] or indirectly through activation of the upstream AMPK kinase TAK1 [342] or through changes in the ATP/ADP ratio [343]. In agreement with the proposal, stress-dependent tubulin hyperacetylation involves H_2_O_2_ activation of the AMPK pathway [246]. Mackeh et al. (2014) showed that AMPK phosphorylation levels increase both during H_2_O_2_ and NaCl stress and that knocking down AMPKα 1/2 by RNAi decreases both basal and stress-induced MT acetylation by 50% when compared to controls [246]. Additionally, STO609, which can inhibit AMPK activity through its inhibition of CaMKK2 [344], inhibits stress-dependent hyperacetylation of MTs [246]. Moreover, stress leads to the appearance of phosphorylated αTAT1, and phosphorylation increases αTAT1 activity [345,346]. Recently, Deb Roy et al. [345] showed, in HeLa cells, that phosphorylated αTAT1 binds to 14-3-3 adapters and accumulates in the cytosol where it can access MTs, whilst the non-phosphorylated αTAT1 form is sequestered inside the nucleus, which allows one to decrease MT acetylation levels [345]. However, phosphorylation of αTAT1 may not be dependent on AMPK activity and could be performed instead by the upstream AMPK kinases. In fact, TAK1 is able to directly catalyze the phosphorylation of αTAT1 at Ser237 and enhance αTAT1 catalytic activity [346].

Other mechanism(s) independent of H_2_O_2_ may also be involved in the regulation of tubulin hyperacetylation during stress because NaCl-induced MT hyperacetylation is only partially reduced by N-acetylcysteine [246]. In fact, tubulin hyperacetylation induced by NaCl stress might also involve slower cellular responses to stress, such as regulation of αTAT-1 expression by the lysine acetyltransferase p300. P300 is a transcriptional coactivator located mainly in the nucleus [347], but, upon NaCl stress, p300 has been shown to translocate to the cytoplasm, where it binds to acetylated MTs [246]. When in the nucleus, p300 inhibits αTAT-1 transcription. Therefore, p300 sequestration in the cytoplasm allows an increased αTAT-1 expression and favors tubulin hyperacetylation. In favor of p300 being a negative regulator of tubulin acetylation is the fact that both basal and stress-induced tubulin acetylation levels increase when p300 is knocked down with siRNA [246]. AMPK might also have a role in the p300-dependent regulation of αTAT1 expression during stress, since AMPK directly phosphorylates and downregulates p300 [338,348,349].

Autophagy is a major pathway through which cells perform waste clearance, and induced autophagy is crucial for sustaining cell homeostasis and, eventually, cell survival during stress conditions. Autophagy is driven by the fusion of lysosomes with autophagosomes to form an autolysosome that degrades misfolded and damaged proteins and organelles [350,351]. The MT network is needed to maintain both the basal and stress-induced autophagic flux, and MTs are involved in the regulation of multiple stages of autophagy [351]. MT α-tubulin hyperacetylation favors cell survival during stress through induction of autophagy [246,331], and α-tubulin acetylated MTs are essential for the autophagosome transport towards a lysosome and their fusion [332]. In fact, when MT α-tubulin hyperacetylation is prevented by knocking down αTAT1 expression using RNAi, there is a decreased starvation-induced autophagy [246]. The main regulator of autophagy is the mammalian target of rapamycin complex 1 (mTORC1), which is one of the main targets of AMPK [338]. Active mTORC1 promotes cell growth and proliferation [352] by catalyzing the direct phosphorylation of two protein targets, EIF4E-binding protein 1 (4E-BP1) and ribosomal protein S6 kinase beta-1 (S6K1), which control the activity of translation eukaryotic initiation factors (eIFs) and eukaryotic elongation factors (eEFs). Active mTORC1 also prevents autophagy by catalyzing the phosphorylation of UNC51-like kinase 1 (ULK1) and its interacting partners, the autophagy related protein 13 (Atg13), focal adhesion kinase (FAK)-family interacting protein of 200 kDa (FIP200), and ATG101, which form the so-called ULK1 complex, thus inhibiting it [353]. Following a cellular stress, mTORC1 is inactivated, which leads to the release of the ULK1 complex, its dephosphorylation, and the subsequent activation of ULK1 kinase activity. ULK1 then catalyzes its own phosphorylation and that of its partners, ATG13 and FIP200, leading to the activation of autophagy. The absence of mTORC1 substrate S6K1 abolishes stress-induced α-tubulin hyperacetylation and disrupts the acetylated MT network, leading to impaired autophagosome-lysosome fusion [354]. S6K1 overexpression restores α-tubulin acetylation and the autophagic flux in stressed S6K1/2-deficient cells. Since these alterations in α-tubulin acetylation were observed in autophagy induced by different stress conditions and in two different cell models, this suggests that S6K1’s role in α-tubulin acetylation is a general phenomenon in stress response.

### 4.2. Dysregulation of Tubulin Acetylation and Disease

Although the functions of tubulin acetylation remain, in most cases, controversial, the association of alterations in this tubulin PTM has long been linked to the development of human diseases.

#### 4.2.1. Neurodegenerative Diseases

Decreased α-tubulin acetylation levels have been observed in several neurodegenerative diseases, including Alzheimer’s disease [355,356], Huntington’s disease [294] Charcot-Marie-Tooth disease [166,357,358,359,360], Amyotrophic lateral sclerosis (ALS) [361], and Parkinson’s disease [295]. These lower levels of acetylated α-tubulin were associated with the defective axonal transport, a MT-dependent process also found in these diseases. In agreement with this proposal, inhibition of HDAC6 restores both normal α-tubulin acetylating levels and axonal transport in these neurodegenerative diseases [295,357,358,359,360]. Moreover, the degeneration of motor and sensory nerves can be prevented by inhibition of HDAC6 when given either prior to or after the onset of symptoms or by deletion of the gene coding for HDAC6, as shown in a mice model of Charcot-Marie-Tooth type 2A disease [360]. The use of HDAC6 inhibitors leads to cognitive deficit improvement, as shown in studies using mice models of Alzheimer’s disease [362,363]. These studies suggest that regulation of α-tubulin acetylation plays a role in the pathogenic mechanisms underlying these neurodegenerative diseases and open the possibility of using HDAC6 inhibition as therapy for these diseases [266,358,364]. However, using inhibitors for HDAC6 has the caveat that other signalling pathways might be affected, since HDAC6 is not specific for α-tubulin and has a diversity of substrates. Additionally, the fact that Atat1^(−/−)^ mice, which have no acetylated α-tubulin, do not develop degenerative phenotypes [288], suggests that α-tubulin acetylation is not the only factor leading to defective axonal transport and the development of these neurodegenerative diseases.

Some insight into the mechanisms by which α-tubulin acetylation affects axonal transport and is involved in the development of different neurodegenerative diseases has been obtained for Parkinson’s and Alzheimer’s disease. Parkinson’s disease features pathogenic intracytoplasmic inclusions (Lewy bodies). α-Synuclein is the main component of Lewy bodies [365], and mutations on the SNCA gene and allele multiplication are linked to familial forms of Parkinson’s disease [366]. α-Synuclein is a MAP involved in regulating MT dynamics [310,367,368]. α-Synuclein binds to both MTs and to tubulin α2β2 tetramer, and the latter interaction induces a structural change in the α-Synuclein polypeptide. This tubulin-induced structural change enables α-Synuclein to promote MT nucleation and to enhance MT growth rate and catastrophe frequency, both in vitro and in vivo. On the contrary, α-Parkinson’s disease-linked α-Synuclein variants do not undergo tubulin-induced folding and cause tubulin aggregation instead of polymerization [368]. Inhibition of SIRT2 leads to an increase in α-tubulin acetylation, a decrease in the formation of α-Synuclein inclusions, and α-Synuclein-mediated neurotoxicity [369]. The most common genetic cause of Parkinson’s disease involves mutations of the Leucine-rich repeat kinase 2 (LRRK2) gene [370,371]. LRRK2 co-localizes with MTs in cells, forming MT-associated LRRK2 filaments, an association enhanced by Parkinson’s disease mutations [372]. Mutant LRRK2 proteins interact directly with three β-tubulin isoforms, TUBB, TUBB4, and TUBB6 [372,373,374] and bind preferentially deacetylated MTs inhibiting axonal transport [295,375]. Association of mutant LRRK2 with MTs is prevented when MT acetylation levels are increased, either by using deacetylase inhibitors or αTAT1, and the HDAC6 inhibitor trichostatin A (TSA) restores axonal transport [295].

Alzheimer’s disease is characterized by two pathological features, involving deposits of misfolded proteins, i.e., extracellular deposition of amyloid-β (Aβ), leading to amyloid plaques and intracellular accumulation of hyperphosphorylated Tau in neurofibrillary tangles (NFTs) [376]. In Alzheimer’s disease studies, conflicting results have been obtained for the levels of acetylated tubulin in postmortem human brain samples. Some studies reported decreased levels of acetylated tubulin in the postmortem brain of Alzheimer’s disease patients [355,356], which could be explained by the higher abundance of HDAC6 found in Alzheimer’s disease [377]. In agreement with these results, studies using tauopathy or Aβ plaque models for Alzheimer’s disease showed that pharmacological inhibition of HDAC6 rescues axonal transport and some features of the Alzheimer’s disease phenotype, e.g., spatial memory deficits, as well as hippocampal synapse loss, possibly through HDAC6 interaction with Tau [362,363,377,378,379,380]. Hyperphosphorylation of Tau and its accumulation has been linked to MT destabilization and cytoskeletal abnormalities and subsequent axonal degeneration [310,378], which regulates axonal function [381]. Tau preferentially binds to acetylated α-tubulin and stabilizes MTs [378]. HDAC6 not just interacts with Tau, but also catalyzes its deacetylation [382], and its inhibition decreases Tau phosphorylation and accumulation [377,380]. However, a direct role of increased tubulin acetylation against Tau toxicity could be established using site-directed α-tubulin Lys40Gln mutagenesis. In fact, acetylation-mimicking α-tubulin was able to rescue Tau-induced MT defects and neuromuscular junction developmental abnormalities [309]. Moreover, α-tubulin acetylation, induced by SIRT2 inhibition, is functionally associated with an improvement of MT dynamics determined by decreased Tau phosphorylation and by increased Tau/tubulin binding [310]. Contrary to the previous studies, other results obtained in postmortem brains suggest that the proportion of acetylated tubulin is increased when normalized for the total MT mass in Alzheimer’s disease [158,308,383]. Increased acetylated tubulin levels were also obtained in human neurons harboring the Alzheimer’s familial APP-V717I mutation [158]. Since these increased acetylated tubulin levels do not correlate with the amounts of HDAC6 or αTAT1, it has been proposed that the reduced MT dynamics associated with impaired tubulin re-tyrosination, which is defective in Alzheimer’s disease, may upregulate the levels of acetylated tubulin, irrespective of the levels of the enzymes regulating tubulin acetylation. [158].

#### 4.2.2. Cancer

In addition to neurological disorders, α-tubulin acetylation has been linked to cancer. Increased levels of MT α-tubulin acetylation have been observed in the head and the neck, pancreatic cancer, and breast cancer [324,384,385]. Higher α-tubulin acetylation levels are a prognostic marker for squamous cell carcinoma of the head and the neck [384]. Increased acetylation of α-tubulin and HDAC6 inhibition enhance autophagy in non-small cell lung cancer [386]. Metastatic breast cancer cells have higher α-tubulin acetylation levels that extend along microtentacles and membrane protrusions in detached cancer cells. These higher α-tubulin acetylation levels are a sufficient cause of metastatic potential and are linked to an increased risk of disease progression and aggressive metastatic behavior [324].

Since α-tubulin acetylation levels are dependent on αTAT1/HDAC6 activities, studies have been made to establish how changes in the activity of these enzymes affect cancer cells, namely, their ability to migrate and metastasize [186,324,387,388,389]. For example, it was suggested that acetylated α-tubulin is a regulator and a marker of epithelial-to-mesenchymal transition (EMT), which has been associated with tumor progression with metastatic expansion. Gu and coworkers (2016) described that increased levels of acetylated α-tubulin were associated with the epithelial morphology and were dramatically decreased during TGF-β-induced EMT as a consequence of the increased activity of HDAC6 by TGF-β [323]. αTAT1 overexpression, which leads to higher α-tubulin acetylation levels, increases the formation of microtentacles and reattachment in a non-metastatic breast cancer cell line [324] and inhibits cancer cell migratory and invasive ability and tumour metastasis on an orthotopic lung cancer animal model [388]. The presence of αTAT1 is needed for extracellular matrix invasion of the highly metastatic MDA-MB-231 breast cancer cell line [186]. The inhibition of HDAC6 by siRNA or HDAC6 inhibitor TSA causes a decreased invasion capacity of MDA-MB-231 cells in a three-dimensional type I collagen matrix [387]. Recently, conflicting results were obtained for several cancer cell lines (the human breast cancer cell lines MCF-7, MDA-MB-231, T1-Luc, a mouse breast cancer cell line, SK-OV-3, a human ovarian cancer cell line, and human mesothelioma MSTO-221 h cells) using selective HDAC6 inhibitors (Tubathian A, Tubastatin A, Tubacin, and Ricolinostat) [389]. HDAC6 inhibition increased acetylation levels of α-tubulin without affecting histone acetylation, but it did not decrease tumor cell growth or block tumor cell migration or invasion. Therefore, although it can be taken from some studies that αTAT1/HDAC6-dependent control of tubulin acetylation levels may have a key role in the regulation of cancer cell migration, invasion, and cancer metastasis, recent contradictory results have shown that further research is needed to clarify the role of α-tubulin acetylation in cancer. Additionally, a recent study using the NCI-60 cancer cell panel showed that, although high levels of acetylated α-tubulin correlate with, they were not required for, taxol cytotoxicity [390].

#### 4.2.3. Cardiac Diseases

Several stresses (e.g., pH, temperature, osmotic stress, mechanical strain, oxidative stress) and also DNA mutations may cause cellular proteins to misfold [391]. Additionally, with ageing, there is a gradual accumulation of both misfolded and damaged proteins associated with failures in the protein quality control of proteins involved in protein maturation, transport, and breakdown. Misfolded proteins can exist as soluble monomers, but they can also associate with each other to form sequentially more complicated protein aggregates, including soluble oligomers, soluble aggregates, and large insoluble protein aggregates called inclusion bodies. All these protein aggregates, including the soluble protein oligomers, can be cytotoxic by stressing the protein degradation machinery, disrupting membrane structure, or sequestering other proteins, and they may induce cell death, a process known as proteotoxicity [392,393].

Proteotoxicity is better known for its role in neurodegenerative diseases, but it has also been associated with cardiac stress disorders and causes heart failure [394,395,396]. In fact, soluble amyloid oligomers have been identified in cardiomyocytes from patients with hypertrophic cardiomyopathy, idiopathic dilated cardiomyopathy, and Becker’s muscular dystrophy, but not in those from healthy controls [397]. In a mouse model of proteinopathy-induced heart failure, α-tubulin was found to be hyperacetylated, whilst other proteins in heart extracts were hypoacetylated. Inhibition of HDAC6 has a protective role against cardiac proteotoxicity, as, for example, when HDAC6 is inhibited, autophagy is promoted, leading to reduced levels of protein aggregates in cardiomyocytes [398].

Changes in acetylated α-tubulin levels are also involved in atrial fibrillation, the most common persistent clinical tachyarrhythmia and an important contributor to cardiovascular morbidity and mortality [399]. Atrial fibrillation induces activation of HADC6, which leads to decreased levels of acetylated α-tubulin, disruption of the cardiomyocyte MT structure, and loss of contractile function [399].

#### 4.2.4. Innate Immunity and Virus Infections

Several DNA and RNA viruses use cytoskeletal networks to efficiently enter, replicate, and exit the host cell while evading host immune responses [400,401]. Tubulin acetylation is involved in every stage of virus infection [402,403,404,405,406]. Regarding viral fusion and entry, it has been shown that the influenza A virus (IAV) induces MT acetylation in epithelial cells through Rho GTPase-mediated downregulation of HDAC6 activity in IAV-infected cells, resulting in increased α-tubulin acetylation [405]. Additionally, the release of virions from infected cells increases with enhanced acetylation of MTs, [405], and HDAC6 inhibits IAV infection [407]. Decreasing acetylation of α-tubulin by HDAC6 overexpression markedly prevents HIV-1 envelope-dependent cell fusion and infection, whilst knockdown of HDAC6 expression or its inhibition enhances HIV-1-cell fusion and HIV-1 infection [402]. Increased α-tubulin acetylation of MTs is also used by numerous DNA and RNA viruses to expedite their intracellular transport to replication sites and to the cell periphery for exit and to enhance their replication [406,408]. HIV-1 has been shown to induce the formation of acetylated and detyrosinated stable MTs early in infection [409]. The human parainfluenza virus type 3 (HPIV3) may form cytoplasmic viral replication sites called inclusion bodies (IBs), in which viral RNA is synthesized. It has been shown that HPIV3 IBs rely on higher cellular α-tubulin acetylation for fusion and maturation [410]. Expression of αTAT1 resulted in the fusion of small IBs into large IBs and effective viral replication whilst overexpressing HDAC6, and SIRT2 profoundly inhibited the fusion of small IBs and viral replication. Recently, it has been shown that vimentin inhibits the formation of HPIV3 IBs and viral replication by decreasing α-tubulin acetylation via enhanced degradation of αTAT1 [411].

Tubulin α-acetylation is also involved in counteracting the innate immune system so that viruses persist in the host. The Epstein-Barr virus (EBV) genome encodes a mitochondrial and ER-localized protein, named BHRF1, which participates in this viral persistence [412]. BHRF1 interacts with αTAT1 to increase MT tubulin α-acetylation levels and, therefore, there is a clustering of autophagosomes and mitochondria near the nucleus. This sequestration of mitochondria inside the autophagosomes leads to mitophagy induction and inhibits the signaling pathway, triggering interferon production.

In summary, altered levels of tubulin acetylation have been implicated in neurodegenerative disorders, cancer, cardiac diseases, and innate immunity. In some cases, e.g., neurodegenerative diseases, the use of inhibitors of the deacetylase HDAC6 has shown some therapeutic effects, namely, rescue of the phenotype of those diseases. However, in most cases, knowledge of the mechanisms underlying the regulation of tubulin acetylation levels and the involvement of tubulin acetylation in the etiology of these diseases is still scarce.

## 5. Concluding Remarks

In the last decades, we have had a clear advancement in our understanding of the role of different PTMS in the variety of MTs functions. However, in the case of acetylation, we still have contradictory observations, and it is still challenging to have a clear picture of the impact of this PTM on MT properties, interactors, and functional modulators.

Tubulin acetylation has been pointed out as a hallmark of stable old MTs. However, the question if it is a cause or a consequence of long-lived MTs has never been clarified. The current view on tubulin acetylation is that this modification alters the mechanical properties of MTs by decreasing inter-protofilament interactions and by allowing MTs to bend and to resist age-related lattice damage caused by multiple interactions with different factors during their existence, thus facilitating MT self-repair [263,264]. However, how this ability of MTs to survive structural damage is translated into specific cellular functions is still controversial, and it is far from being elucidated. One of the major difficulties in establishing the cellular functions of tubulin acetylation relies on the fact that most of the approaches used to address these questions have relied on the manipulation of acetyltransferases and deacetylases levels and activities. Unfortunately, our knowledge about the functions and possibly complex regulation in vivo of these enzymes is limited. In fact, it is already established that these enzymes have other substrates besides tubulin, especially the deacetylases. This raises the hypothesis that their impact on MT structure and function is not just a direct effect, but it may also be due to indirect effects through the pathways where their other substrates are players. In this scenario, alterations in the activities of acetytransferases/deacetylases may lead to diverse patterns of acetylated substrates, resulting in different functional outcomes. This complexity will be expanded if the cell type and microenvironment are included in the analysis. For example, studies have shown that, in cell culture, changing the adhesion substrates [157] or cell plating number causes a change in the acetylation pattern of MTs.

Moreover, although all eukaryotic cells have a cytoskeleton, the organization and the abundance of distinct components of the cytoskeleton varies from cell type to cell type. In fact, there is evidence that other cytoskeleton components may regulate tubulin acetylation. For example, Rho GTPases, critical molecules in actin cytoskeleton reorganization, mediate the downregulation of HDAC6 [405,413], and vimentin, an intermediate filaments protein, decreases tubulin acetylation levels via αTAT1 degradation [411]. Illustrating this are the heterogeneous tubulin acetylation patterns in different cell types and also in distinct cancer cells from different origins [390]. Additionally, the consequences of tubulin acetylation will be modulated by the content (identity and abundance) of MAPs and motor proteins in each cell type. These proteins will be able to read, remodel, and function according to tubulin acetylation patterns, as proposed by the tubulin code model [142,161]. This will have direct consequences in generating distinct cell responses, upon changes in tubulin acetylation/deacetylation, and in response to challenges and specific stress situations that cells have to cope with within their physiological microenvironment.

Furthermore, we are also probably losing information because most works only analyze the impact of an isolated PTM at a time. It is probable that tubulin acetylation works in close crosstalk with other tubulin PTMs. For example, the fact that methylation also occurs in Lys40 and competes with acetylation for this Lys residue [155]. The crosstalk between different PTMs may create a high number of combinatorial patterns, and in certain situations, these patterns will create redundancy, causing difficulties in the analysis of the phenotypes. This indicates that future approaches to studying acetylation roles require more integrated analysis that incorporates, whenever possible, the ability of cells, in the context of their intrinsic features (e.g., cytoskeleton organization, metabolic activity, migration, and proliferation), to respond to challenges and their physical/mechanical and physiological microenvironments. More attention should be paid to tubulin acetylation’s role in generating different cellular MT subset arrays that may create specific local and temporal organization, impacting the three-dimensional positioning of organelles. For example, not much is known about the fact that MTs nucleated by the Golgi apparatus are rapidly acetylated when newly polymerized [414].

Intriguingly, no data exist concerning the functional relationship between tubulin cofactors and tubulin PTMs, such as acetylation. In fact, these proteins play critical roles in tubulin heterodimer assembly/dissociation, tubulin degradation, as well as in maintaining tubulin pools and tubulin heterodimers recycling/degradation by controlling their native structure’s quality [5,13]. Therefore, these proteins are potential candidates to assist in the remodeling of PTMs patterns. Tubulin acetylation is proposed to facilitate the self-repair of mechanically damaged MT, a process that should require the removal of damaged tubulin dimers and the incorporation of free ones. Consequently, mechanisms regulating this repair process should probably involve tubulin cofactors activities, for example, the control of the tubulin pool of free heterodimers competent to polymerize, and future research efforts should cover their involvement.

## Figures and Tables

**Table 1:** Brief summary of α and β-tubulin acetylation functions.

Acetylated Tubulin and Amino Acid Residue	Acetylases/Deacetylases	Structural Localization	Functions	References
α-tubulin lysine 40 (Lys40) residue	αTAT1/HDAC6 and SIRT2	Unstructured loop of α-tubulin of polymerized MTs.	**Mechanical properties of MTs and prevention from mechanical aging** - Establishing salt bridges between adjacent α-tubulins, leading to a rearrangement in the inter-protofilament angle. - Reducing interactions between protofilaments, potentially promoting protofilament sliding and enhancing MT flexibility and controlling the protofilament number. - Modulation of lateral, but not longitudinal, interactions of the protofilaments. - Avoiding long-lived MTs to lose their rigidity due to repetitive bending and stopping MT breakage and extending the MTs’ lifespan.	[157,167,187,231,237,257,259,269]
			**Regulation of MT dynamics** - Self-assembly rate of acetylated tubulin was much slower than that of deacetylated tubulin. - No differences were found in polymerization rates for acetylated and deacetylated tubulin - When the measurements were made with the MTs already formed - acetylated MTs had a disassembly rate threefold faster than deacetylated MTs	[157,260,263,264]
			**Acetylation in centrioles** - Allow centrioles to bear more mechanical stress and rendering them more flexible.	[282]
			**Cilia assembly** - Lys40 acetylation is the most abundant acetylation site in cilia MTs - It is controversial that Lys40 acetylation is critical for correct cilia assembly and structure maintenance and stability. This may depend on cell type. - Controversial if it is required for sperm motility - Controversial in controlling ciliary motor proteins	[145,165,167,187,244,273,277,278,279,280]
			**Cargo transport** - Cytoplasmic MT acetylation is more heterogeneous than that of cilia and centrioles. - The abundance and distribution patterns vary according to cell type, cell life cycle, and cell region; neurons illustrate this complexity. - A relationship between the MT acetylation status and the binding of molecular motors dynein and kinesin, especially in neurons, has been suggested. - Regulation of intracellular trafficking	[166,205,253,279,280,284,285,286,287,288,292,293,294,295,296,297,298,299]
			**Cell division** - The acetylation of α-tubulin Lys40 is highly abundant in the mitotic spindle, midbody, and kinetochore MTs. - It is controversial that cell cycle progression is affected by MT acetylation/deacetylation. - Enzymes αTAT1, HDAC6, and SIRT2 present a regulation dependent on the cell cycle.	[166,167,235,270,311,312,316,317,318,319]
			**Cell shape and migration** - MT acetylation is required for change in platelets’ shapes. - Tubulin acetylation in migration is involved in intracellular rearrangement of MTs accompanied by organelles re-localization. - It is controversial that loss of either HDAC6 or αTAT1 is associated with an increase in the number of focal adhesions. - It is proposed that MT acetylation is critical for the fine tuning of the mechanosensitive cell adhesion and migration. - Acetylation of MTs is critical for the penetrative capacity of cells undergoing radial intercalation in epithelia.	[186,192,204,237,259,320,321,322,323,324,325,326,327]
			**Stress response and autophagy** - Increased levels of MT acetylation have been found in cells exposed to several cellular stresses, and this leads to increased cell survival. - MT α-tubulin hyperacetylation favors cell survival during stress through the induction of autophagy.	[227,246,331,332]
β-tubulin Lys252 residue	SAN/?	Interface between α/β-tubulins in the heterodimer	**Stabilization of tubulin heterodimer** - Affect the α/β tubulin interaction with impact in MT polymerization - Low levels of San acetylase, after cold-shock depolymerization, leads to MT depolymerization at a normal rate, but MT regrowth is faster.	[176]
α-tubulin Lys394 residue	?/HDAC6	Interface of the α/β-tubulin heterodimer in the MT surface, a region that suffers a conformational change as the dimers are added to the MT	**Microtubule polymerization** - This is required for heterodimer incorporation during MT assembly.	[135,175,330]

## Data Availability

Not applicable.

## References

[1] Liu Y., Tavana O., Gu W. (2019). p53 modifications: Exquisite decorations of the powerful guardian. J. Mol. Cell Biol..

[2] Millán-Zambrano G., Burton A., Bannister A.J., Schneider R. (2022). Histone post-translational modifications—Cause and consequence of genome function. Nat. Rev. Genet..

[3] Wloga D., Joachimiak E., Fabczak H. (2017). Tubulin Post-Translational Modifications and Microtubule Dynamics. Int. J. Mol. Sci..

[4] Gudimchuk N.B., McIntosh J.R. (2021). Regulation of microtubule dynamics, mechanics and function through the growing tip. Nat. Rev. Mol. Cell Biol..

[5] Gonçalves J., Tavares A., Carvalhal S., Soares H. (2010). Revisiting the tubulin folding pathway: New roles in centrosomes and cilia. Biomol. Concepts.

[6] Cowan N.J., Lewis S.A. (2001). Type II chaperonns, prefoldin, and the tubulin-specific chaperones. Adv. Protein Chem..

[7] Lopez T., Dalton K., Frydman J. (2015). The Mechanism and Function of Group II Chaperonins. J. Mol. Biol..

[8] Lewis S.A., Tian G., Cowan N.J. (1997). The α- and β-tubulin folding pathways. Trends Cell Biol..

[9] Lopez-Fanarraga M., Avila J., Guasch A., Coll M., Zabala J.C. (2001). Review: Postchaperonin Tubulin Folding Cofactors and Their Role in Microtubule Dynamics. J. Struct. Biol..

[10] Kortazar D., Fanarraga M., Carranza G., Bellido J., Villegas J., Avila J., Zabala J. (2007). Role of cofactors B (TBCB) and E (TBCE) in tubulin heterodimer dissociation. Exp. Cell Res..

[11] Voloshin O., Gocheva Y., Gutnick M., Movshovich N., Bakhrat A., Baranes-Bachar K., Bar-Zvi D., Parvari R., Gheber L., Raveh D. (2010). Tubulin chaperone E binds microtubules and proteasomes and protects against misfolded protein stress. Cell. Mol. Life Sci..

[12] Desai A., Mitchison T.J. (1997). Microtubule Polymerization Dynamics. Annu. Rev. Cell Dev. Biol..

[13] Serna M., Zabala J.C. (2016). Tubulin Folding and Degradation. eLS.

[14] Nolasco S., Bellido J., Serna M., Carmona B., Soares H., Zabala J.C. (2021). Colchicine Blocks Tubulin Heterodimer Recycling by Tubulin Cofactors TBCA, TBCB, and TBCE. Front. Cell Dev. Biol..

[15] Roll-Mecak A. (2020). The Tubulin Code in Microtubule Dynamics and Information Encoding. Dev. Cell.

[16] Camelo C., Peneda C., Carmona B., Soarets H., Choi S. (2016). TBCC. Encyclopedia of Signaling Molecules.

[17] Lomakin A.J., Semenova I., Zaliapin I., Kraikivski P., Nadezhdina E., Slepchenko B.M., Akhmanova A., Rodionov V. (2009). CLIP-170-Dependent Capture of Membrane Organelles by Microtubules Initiates Minus-End Directed Transport. Dev. Cell.

[18] Lomakin A.J., Kraikivski P., Semenova I., Ikeda K., Zaliapin I., Tirnauer J.S., Akhmanova A., Rodionov V. (2011). Stimulation of the CLIP-170–dependent capture of membrane organelles by microtubules through fine tuning of microtubule assembly dynamics. Mol. Biol. Cell.

[19] Kanfer G., Peterka M., Arzhanik V.K., Drobyshev A.L., Ataullakhanov F.I., Volkov V.A., Kornmann B. (2017). CENP-F couples cargo to growing and shortening microtubule ends. Mol. Biol. Cell.

[20] Seetharaman S., Etienne-Manneville S. (2019). Microtubules at focal adhesions—A double-edged sword. J. Cell Sci..

[21] Stehbens S., Wittmann T. (2012). Targeting and transport: How microtubules control focal adhesion dynamics. J. Cell Biol..

[22] Stehbens S.J., Paszek M., Pemble H., Ettinger A., Gierke S., Wittmann T. (2014). CLASPs link focal-adhesion-associated microtubule capture to localized exocytosis and adhesion site turnover. Nature.

[23] Kopf A., Kiermaier E. (2021). Dynamic Microtubule Arrays in Leukocytes and Their Role in Cell Migration and Immune Synapse Formation. Front. Cell Dev. Biol..

[24] Seetharaman S., Etienne-Manneville S. (2020). Cytoskeletal Crosstalk in Cell Migration. Trends Cell Biol..

[25] Mayor R., Etienne-Manneville S. (2016). The front and rear of collective cell migration. Nat. Rev. Mol. Cell Biol..

[26] Bayless K.J., Johnson G.A. (2011). Role of the Cytoskeleton in Formation and Maintenance of Angiogenic Sprouts. J. Vasc. Res..

[27] Hohmann T., Dehghani F. (2019). The Cytoskeleton—A Complex Interacting Meshwork. Cells.

[28] Bornens M. (2008). Organelle positioning and cell polarity. Nat. Rev. Mol. Cell Biol..

[29] Budovsky A., Fraifeld V.E., Aronov S. (2010). Linking cell polarity, aging and rejuvenation. Biogerontology.

[30] Soares H., Marinho H.S., Real C., Antunes F. (2013). Cellular polarity in aging: Role of redox regulation and nutrition. Genes Nutr..

[31] Vasileva E., Citi S. (2018). The role of microtubules in the regulation of epithelial junctions. Tissue Barriers.

[32] Matis M. (2020). The Mechanical Role of Microtubules in Tissue Remodeling. Bioessays.

[33] Singh A., Saha T., Begemann I., Ricker A., Nüsse H., Thorn-Seshold O., Klingauf J., Galic M., Matis M. (2018). Polarized microtubule dynamics directs cell mechanics and coordinates forces during epithelial morphogenesis. Nat. Cell Biol..

[34] Ghalloussi D., Dhenge A., Bergmeier W. (2019). New insights into cytoskeletal remodeling during platelet production. J. Thromb. Haemost..

[35] Patel S.R., Richardson J.L., Schulze H., Kahle E., Galjart N., Drabek K., Shivdasani R.A., Hartwig J.H., Italiano J.J.E. (2005). Differential roles of microtubule assembly and sliding in proplatelet formation by megakaryocytes. Blood.

[36] Thon J.N., Macleod H., Begonja A.J., Zhu J., Lee K.-C., Mogilner A., Hartwig J.H., Jr J.E.I. (2012). Microtubule and cortical forces determine platelet size during vascular platelet production. Nat. Commun..

[37] Sánchez I., Dynlacht B.D. (2016). Cilium assembly and disassembly. Nat. Cell Biol..

[38] Pitaval A., Senger F., Letort G., Gidrol X., Guyon L., Sillibourne J., Théry M. (2017). Microtubule stabilization drives 3D centrosome migration to initiate primary ciliogenesis. J. Cell Biol..

[39] van der Vaart B., Akhmanova A., Straube A. (2009). Regulation of microtubule dynamic instability. Biochem. Soc. Trans..

[40] Bodakuntla S., Jijumon A., Villablanca C., Gonzalez-Billault C., Janke C. (2019). Microtubule-Associated Proteins: Structuring the Cytoskeleton. Trends Cell Biol..

[41] Kapoor T.M. (2017). Metaphase Spindle Assembly. Biology.

[42] Starr D.A. (2009). A nuclear-envelope bridge positions nuclei and moves chromosomes. J. Cell Sci..

[43] Dogterom M., Kerssemakers J.W.J., Romet-Lemonne G., E Janson M. (2005). Force generation by dynamic microtubules. Curr. Opin. Cell Biol..

[44] Kimura A., Onami S. (2010). Modeling Microtubule-Mediated Forces and Centrosome Positioning in *Caenorhabditis elegans* Embryos. Methods Cell Biol..

[45] Burakov A., Nadezhdina E., Slepchenko B., Rodionov V. (2003). Centrosome positioning in interphase cells. J. Cell Biol..

[46] Zhu J., Burakov A., Rodionov V., Mogilner A. (2010). Finding the Cell Center by a Balance of Dynein and Myosin Pulling and Microtubule Pushing: A Computational Study. Mol. Biol. Cell.

[47] Wu J., Misra G., Russell R.J., Ladd A.J.C., Lele T.P., Dickinson R.B. (2011). Effects of dynein on microtubule mechanics and centrosome positioning. Mol. Biol. Cell.

[48] Tanimoto H., Sallé J., Dodin L., Minc N. (2018). Physical forces determining the persistency and centring precision of microtubule asters. Nat. Phys..

[49] Odell J., Sikirzhytski V., Tikhonenko I., Cobani S., Khodjakov A., Koonce M. (2019). Force balances between interphase centrosomes as revealed by laser ablation. Mol. Biol. Cell.

[50] Daga R.R., Yonetani A., Chang F. (2006). Asymmetric Microtubule Pushing Forces in Nuclear Centering. Curr. Biol..

[51] Tran P., Marsh L., Doye V., Inoué S., Chang F. (2001). A Mechanism for Nuclear Positioning in Fission Yeast Based on Microtubule Pushing. J. Cell Biol..

[52] Zhao T., Graham O.S., Raposo A., Johnston D.S. (2012). Growing Microtubules Push the Oocyte Nucleus to Polarize the *Drosophila* Dorsal-Ventral Axis. Science.

[53] Letort G., Nedelec F., Blanchoin L., Théry M. (2016). Centrosome centering and decentering by microtubule network rearrangement. Mol. Biol. Cell.

[54] Théry M., Racine V., Piel M., Pépin A., Dimitrov A., Chen Y., Sibarita J.-B., Bornens M. (2006). Anisotropy of cell adhesive microenvironment governs cell internal organization and orientation of polarity. Proc. Natl. Acad. Sci. USA.

[55] Komarova Y., De Groot C.O., Grigoriev I., Gouveia S.M., Munteanu E.L., Schober J.M., Honnappa S., Buey R.M., Hoogenraad C.C., Dogterom M. (2009). Mammalian end binding proteins control persistent microtubule growth. J. Cell Biol..

[56] Akhmanova A., Steinmetz M.O. (2008). Tracking the ends: A dynamic protein network controls the fate of microtubule tips. Nat. Rev. Mol. Cell Biol..

[57] Lansbergen G., Akhmanova A. (2006). Microtubule Plus End: A Hub of Cellular Activities. Traffic.

[58] Honnappa S., Gouveia S., Weisbrich A., Damberger F., Bhavesh N.S., Jawhari H., Grigoriev I., van Rijssel F.J., Martinez-Buey R., Lawera A. (2009). An EB1-Binding Motif Acts as a Microtubule Tip Localization Signal. Cell.

[59] Vitre B., Coquelle F.M., Heichette C., Garnier C., Chrétien D., Arnal I. (2008). EB1 regulates microtubule dynamics and tubulin sheet closure in vitro. Nat. Cell Biol..

[60] Bieling P., Laan L., Schek H., Munteanu E.L., Sandblad L., Dogterom M., Brunner D., Surrey T. (2007). Reconstitution of a microtubule plus-end tracking system in vitro. Nature.

[61] Maurer S.P., Fourniol F.J., Bohner G., Moores C.A., Surrey T. (2012). EBs Recognize a Nucleotide-Dependent Structural Cap at Growing Microtubule Ends. Cell.

[62] Seetapun D., Castle B.T., McIntyre A.J., Tran P.T., Odde D.J. (2012). Estimating the Microtubule GTP Cap Size In Vivo. Curr. Biol..

[63] Zhang R., Alushin G.M., Brown A., Nogales E. (2015). Mechanistic Origin of Microtubule Dynamic Instability and Its Modulation by EB Proteins. Cell.

[64] Duellberg C., Cade N.I., Holmes D., Surrey T. (2016). The size of the EB cap determines instantaneous microtubule stability. Elife.

[65] Kumar P., Lyle K.S., Gierke S., Matov A., Danuser G., Wittmann T. (2009). GSK3β phosphorylation modulates CLASP–microtubule association and lamella microtubule attachment. J. Cell Biol..

[66] Lansbergen G., Grigoriev I., Mimori-Kiyosue Y., Ohtsuka T., Higa S., Kitajima I., Demmers J., Galjart N., Houtsmuller A.B., Grosveld F. (2006). CLASPs Attach Microtubule Plus Ends to the Cell Cortex through a Complex with LL5β. Dev. Cell.

[67] Galjart N. (2005). CLIPs and CLASPs and cellular dynamics. Nat. Rev. Mol. Cell Biol..

[68] Walczak C.E., Gayek S., Ohi R. (2013). Microtubule-Depolymerizing Kinesins. Annu. Rev. Cell Dev. Biol..

[69] Cassimeris L. (2002). The oncoprotein 18/stathmin family of microtubule destabilizers. Curr. Opin. Cell Biol..

[70] Gupta K.K., Li C., Duan A., Alberico E.O., Kim O.V., Alber M.S., Goodson H.V. (2013). Mechanism for the catastrophe-promoting activity of the microtubule destabilizer Op18/stathmin. Proc. Natl. Acad. Sci. USA.

[71] Roostalu J., Surrey T. (2017). Microtubule nucleation: Beyond the template. Nat. Rev. Mol. Cell Biol..

[72] McNally F.J., Vale R.D. (1993). Identification of katanin, an ATPase that severs and disassembles stable microtubules. Cell.

[73] Evans K.J., Gomes E., Reisenweber S.M., Gundersen G.G., Lauring B.P. (2005). Linking axonal degeneration to microtubule remodeling by Spastin-mediated microtubule severing. J. Cell Biol..

[74] Roll-Mecak A., Vale R.D. (2005). The *Drosophila* Homologue of the Hereditary Spastic Paraplegia Protein, Spastin, Severs and Disassembles Microtubules. Curr. Biol..

[75] Mukherjee S., Valencia J.D.D., Stewman S., Metz J., Monnier S., Rath U., Asenjo A.B., Charafeddine R.A., Sosa H.J., Ross J.L. (2012). Human Fidgetin is a microtubule severing the enzyme and minus-end depolymerase that regulates mitosis. Cell Cycle.

[76] Kuo Y.-W., Howard J. (2020). Cutting, Amplifying, and Aligning Microtubules with Severing Enzymes. Trends Cell Biol..

[77] McNally F.J., Roll-Mecak A. (2018). Microtubule-severing enzymes: From cellular functions to molecular mechanism. J. Cell Biol..

[78] Zhang D., Grode K.D., Stewman S.F., Diaz-Valencia J.D., Liebling E., Rath U., Riera T., Currie J.D., Buster D.W., Asenjo A.B. (2011). *Drosophila* katanin is a microtubule depolymerase that regulates cortical-microtubule plus-end interactions and cell migration. Nat. Cell Biol..

[79] E Mains P., Kemphues K.J., A Sprunger S., A Sulston I., Wood W.B. (1990). Mutations affecting the meiotic and mitotic divisions of the early *Caenorhabditis elegans* embryo. Genetics.

[80] Ahmad F., Yu W., McNally F.J., Baas P.W. (1999). An Essential Role for Katanin in Severing Microtubules in the Neuron. J. Cell Biol..

[81] Kapitein L.C., Hoogenraad C.C. (2015). Building the Neuronal Microtubule Cytoskeleton. Neuron.

[82] Baas P.W., Rao A.N., Matamoros A.J., Leo L. (2016). Stability properties of neuronal microtubules. Cytoskeleton.

[83] Lohret T.A., McNally F.J., Quarmby L.M. (1998). A Role for Katanin-mediated Axonemal Severing during *Chlamydomonas* Deflagellation. Mol. Biol. Cell.

[84] Vemu A., Szczesna E., Zehr E.A., Spector J.O., Grigorieff N., Deaconescu A.M., Roll-Mecak A. (2018). Severing enzymes amplify microtubule arrays through lattice GTP-tubulin incorporation. Science.

[85] Komarova Y.A., Akhmanova A.S., Kojima S.-I., Galjart N., Borisy G.G. (2002). Cytoplasmic linker proteins promote microtubule rescue in vivo. J. Cell Biol..

[86] Chen J., Kanai Y., Cowan N.J., Hirokawa N. (1992). Projection domains of MAP2 and tau determine spacings between microtubules in dendrites and axons. Nature.

[87] Harada A., Oguchi K., Okabe S., Kuno J., Terada S., Ohshima T., Sato-Yoshitake R., Takei Y., Noda T., Hirokawa N. (1994). Altered microtubule organization in small-calibre axons of mice lacking tau protein. Nature.

[88] Shahpasand K., Uemura I., Saito T., Asano T., Hata K., Shibata K., Toyoshima Y., Hasegawa M., Hisanaga S.-I. (2012). Regulation of Mitochondrial Transport and Inter-Microtubule Spacing by Tau Phosphorylation at the Sites Hyperphosphorylated in Alzheimer’s Disease. J. Neurosci..

[89] Fourniol F.J., Sindelar C.V., Amigues B., Clare D.K., Thomas G., Perderiset M., Francis F., Houdusse A., Moores C.A. (2010). Template-free 13-protofilament microtubule–MAP assembly visualized at 8 Å resolution. J. Cell Biol..

[90] Hooikaas P.J., Martin M., Mühlethaler T., Kuijntjes G.-J., Peeters C.A., Katrukha E.A., Ferrari L., Stucchi R., Verhagen D.G., van Riel W.E. (2019). MAP7 family proteins regulate kinesin-1 recruitment and activation. J. Cell Biol..

[91] Subramanian R., Wilson-Kubalek E.M., Arthur C.P., Bick M.J., Campbell E.A., Darst S.A., Milligan R.A., Kapoor T.M. (2010). Insights into Antiparallel Microtubule Crosslinking by PRC1, a Conserved Nonmotor Microtubule Binding Protein. Cell.

[92] Bieling P., Telley I.A., Surrey T. (2010). A Minimal Midzone Protein Module Controls Formation and Length of Antiparallel Microtubule Overlaps. Cell.

[93] Ichikawa M., Bui K.H. (2018). Microtubule Inner Proteins: A Meshwork of Luminal Proteins Stabilizing the Doublet Microtubule. Bioessays.

[94] Khalifa A.A.Z., Ichikawa M., Dai D., Kubo S., Black C.S., Peri K., McAlear T.S., Veyron S., Yang S.K., Vargas J. (2020). The inner junction complex of the cilia is an interaction hub that involves tubulin post-translational modifications. Elife.

[95] Ma M., Stoyanova M., Rademacher G., Dutcher S.K., Brown A., Zhang R. (2019). Structure of the Decorated Ciliary Doublet Microtubule. Cell.

[96] Goodson H.V., Jonasson E.M. (2018). Microtubules and Microtubule-Associated Proteins. Cold Spring Harb. Perspect. Biol..

[97] Dumont E.L.P., Do C., Hess H. (2015). Molecular wear of microtubules propelled by surface-adhered kinesins. Nat. Nanotechnol..

[98] Triclin S., Inoue D., Gaillard J., Htet Z.M., DeSantis M.E., Portran D., Derivery E., Aumeier C., Schaedel L., John K. (2021). Self-repair protects microtubules from destruction by molecular motors. Nat. Mater..

[99] Janke C. (2014). The tubulin code: Molecular components, readout mechanisms, and functions. J. Cell Biol..

[100] Wloga D., Joachimiak E., Louka P., Gaertig J. (2016). Posttranslational Modifications of Tubulin and Cilia. Cold Spring Harb. Perspect. Biol..

[101] Ludueña R.F. (2013). A Hypothesis on the Origin and Evolution of Tubulin. Int. Rev. Cell Mol. Biol..

[102] Breuss M.W., Leca I., Gstrein T., Hansen A.H., Keays D.A. (2017). Tubulins and brain development—The origins of functional specification. Mol. Cell. Neurosci..

[103] Kemphues K.J., Raff E.C., Raff R.A., Kaufman T.C. (1980). Mutation in a testis-specific β-tubulin in *Drosophila*: Analysis of its effects on meiosis and map location of the gene. Cell.

[104] Nielsen M.G., Turner F., Hutchens J.A., Raff E.C. (2001). Axoneme-specific β-tubulin specialization: A Conserved C-Terminal Motif Specifies the Central Pair. Curr. Biol..

[105] Fukushige T., Siddiqui Z., Chou M., Culotti J., Gogonea C., Siddiqui S., Hamelin M. (1999). MEC-12, an alpha-tubulin required for touch sensitivity in *C. elegans*. J. Cell Sci..

[106] Savage C., Hamelin M., Culotti J.G., Coulson A., Albertson D.G., Chalfie M. (1989). mec-7 is a beta-tubulin gene required for the production of 15-protofilament microtubules in *Caenorhabditis elegans*. Genes Dev..

[107] Schwer H.D., Lecine P., Tiwari S., E Italiano J., Hartwig J.H., A Shivdasani R. (2001). A lineage-restricted and divergent β-tubulin isoform is essential for the biogenesis, structure and function of blood platelets. Curr. Biol..

[108] Janke C., Magiera M.M. (2020). The tubulin code and its role in controlling microtubule properties and functions. Nat. Rev. Mol. Cell Biol..

[109] Song Y., Brady S.T. (2014). Post-translational modifications of tubulin: Pathways to functional diversity of microtubules. Trends Cell Biol..

[110] Fourest-Lieuvin A., Peris L., Gache V., Garcia-Saez I., Juillan-Binard C., Lantez V., Job D. (2006). Microtubule Regulation in Mitosis: Tubulin Phosphorylation by the Cyclin-dependent Kinase Cdk1. Mol. Biol. Cell.

[111] Ori-McKenney K.M., McKenney R.J., Huang H.H., Li T., Meltzer S., Jan L.Y., Vale R.D., Wiita A.P., Jan Y.N. (2016). Phosphorylation of β-Tubulin by the Down Syndrome Kinase, Minibrain/DYRK1a, Regulates Microtubule Dynamics and Dendrite Morphogenesis. Neuron.

[112] Ludueña R.F., Zimmermann H.-P., Little M. (1988). Identification of the phosphorylated β-tubulin isotype in differentiated neuroblastoma cells. FEBS Lett..

[113] Peters J.D., Furlong M.T., Asai D.J., Harrison M.L., Geahlen R.L. (1996). Syk, Activated by Cross-linking the B-cell Antigen Receptor, Localizes to the Cytosol Where It Interacts with and Phosphorylates α-Tubulin on Tyrosine. J. Biol. Chem..

[114] Akiyama T., Kadowaki T., Nishida E., Kadooka T., Ogawara H., Fukami Y., Sakai H., Takaku F., Kasuga M. (1986). Substrate specificities of tyrosine-specific protein kinases toward cytoskeletal proteins in vitro. J. Biol. Chem..

[115] Matten W.T., Aubry M., West J., Maness P.F. (1990). Tubulin is phosphorylated at tyrosine by pp60c-src in nerve growth cone membranes. J. Cell Biol..

[116] Park I.Y., Powell R.T., Tripathi D.N., Dere R., Ho T.H., Blasius T.L., Chiang Y.-C., Davis I.J., Fahey C.C., Hacker K.E. (2016). Dual Chromatin and Cytoskeletal Remodeling by SETD2. Cell.

[117] Chin H.G., Esteve P.-O., Ruse C., Lee J., Schaus S.E., Pradhan S., Hansen U. (2020). The microtubule-associated histone methyltransferase SET8, facilitated by transcription factor LSF, methylates α-tubulin. J. Biol. Chem..

[118] Ozols J., Caron J.M. (1997). Posttranslational modification of tubulin by palmitoylation: II. Identification of sites of palmitoylation. Mol. Biol. Cell.

[119] Song Y., Kirkpatrick L.L., Schilling A.B., Helseth D.L., Chabot N., Keillor J.W., Johnson G.V., Brady S.T. (2013). Transglutaminase and Polyamination of Tubulin: Posttranslational Modification for Stabilizing Axonal Microtubules. Neuron.

[120] Hallak M.E., Rodriguez J., Barra H., Caputto R. (1977). Release of tyrosine from tyrosinated tubulin. Some common factors that affect this process and the assembly of tubulin. FEBS Lett..

[121] Barra H.S., Arce C.A., Rodriguez J.A., Caputto R. (1973). Incorporation of phenylalanine as a single unit into rat brain protein: Reciprocal inhibition by phenylalanine and tyrosine of their respective incorporations. J. Neurochem..

[122] Arce C.A., Rodriguez J.A., Barra H.S., Caputto R. (1975). Incorporation of l-Tyrosine, l-Phenylalanine and l-3,4-Dihydroxyphenylalanine as Single Units into Rat Brain Tubulin. JBIC J. Biol. Inorg. Chem..

[123] Redeker V., Levilliers N., Schmitter J.-M., Le Caer J.-P., Rossier J., Adoutte A., Bré M.-H. (1994). Polyglycylation of Tubulin: A Posttranslational Modification in Axonemal Microtubules. Science.

[124] Bre M., Redeker V., Quibell M., Darmanaden-Delorme J., Bressac C., Cosson J., Huitorel P., Schmitter J., Rossler J., Johnson T. (1996). Axonemal tubulin polyglycylation probed with two monoclonal antibodies: Widespread evolutionary distribution, appearance during spermatozoan maturation and possible function in motility. J. Cell Sci..

[125] Eddé B., Rossier J., Le Caer J.-P., Desbruyères E., Gros F., Denoulet P. (1990). Posttranslational Glutamylation of α-tubulin. Science.

[126] E Alexander J., Hunt D.F., Lee M.K., Shabanowitz J., Michel H., Berlin S.C., MacDonald T.L., Sundberg R.J., I Rebhun L., Frankfurter A. (1991). Characterization of posttranslational modifications in neuron-specific class III beta-tubulin by mass spectrometry. Proc. Natl. Acad. Sci. USA.

[127] Rüdiger M., Plessman U., Klöppel K.-D., Wehland J., Weber K. (1992). Class II tubulin, the major brain β tubulin isotype is polyglutamylated on glutamic acid residue 435. FEBS Lett..

[128] Ren Y., Zhao J., Feng J. (2003). Parkin Binds to α/β Tubulin and Increases their Ubiquitination and Degradation. J. Neurosci..

[129] Wang Q., Peng Z., Long H., Deng X., Huang K. (2019). Poly-ubiquitylation of α-tubulin at K304 is required for flagellar disassembly in *Chlamydomonas*. J. Cell Sci..

[130] Rosas-Acosta G., Russell W.K., Deyrieux A., Russell D.H., Wilson V.G. (2005). A Universal Strategy for Proteomic Studies of SUMO and Other Ubiquitin-like Modifiers. Mol. Cell. Proteom..

[131] Paturle L., Wehland J., Margolis R.L., Job D. (1989). Complete separation of tyrosinated, detyrosinated, and nontyrosinatable brain tubulin subpopulations using affinity chromatography. Biochemistry.

[132] Paturle-Lafanechere L., Edde B., Denoulet P., Van Dorsselaer A., Mazarguil H., Le Caer J.P., Wehland J., Job D. (1991). Characterization of a major brain tubulin variant which cannot be tyrosinated. Biochemistry.

[133] Aillaud C., Bosc C., Saoudi Y., Denarier E., Peris L., Sago L., Taulet N., Cieren A., Tort O., Magiera M.M. (2016). Evidence for new C-terminally truncated variants of α- and β-tubulins. Mol. Biol. Cell.

[134] Hansen B.K., Gupta R., Baldus L., Lyon D., Narita T., Lammers M., Choudhary C., Weinert B.T. (2019). Analysis of human acetylation stoichiometry defines mechanistic constraints on protein regulation. Nat. Commun..

[135] Liu N., Xiong Y., Li S., Ren Y., He Q., Gao S., Zhou J., Shui W. (2015). New HDAC6-mediated deacetylation sites of tubulin in the mouse brain identified by quantitative mass spectrometry. Sci. Rep..

[136] Liu N., Xiong Y., Ren Y., Zhang L., He X., Wang X., Liu M., Li D., Shui W., Zhou J. (2015). Proteomic Profiling and Functional Characterization of Multiple Post-Translational Modifications of Tubulin. J. Proteome Res..

[137] Lundby A., Lage K., Weinert B.T., Bekker-Jensen D.B., Secher A., Skovgaard T., Kelstrup C.D., Dmytriyev A., Choudhary C., Lundby C. (2012). Proteomic Analysis of Lysine Acetylation Sites in Rat Tissues Reveals Organ Specificity and Subcellular Patterns. Cell Rep..

[138] Weinert B.T., Wagner S.A., Horn H., Henriksen P., Liu W.R., Olsen J.V., Jensen L.J., Choudhary C. (2011). Proteome-Wide Mapping of the *Drosophila* Acetylome Demonstrates a High Degree of Conservation of Lysine Acetylation. Sci. Signal..

[139] Choudhary C., Kumar C., Gnad F., Nielsen M.L., Rehman M., Walther T.C., Olsen J.V., Mann M. (2009). Lysine Acetylation Targets Protein Complexes and Co-Regulates Major Cellular Functions. Science.

[140] Piroli G.G., Manuel A.M., Walla M.D., Jepson M.J., Brock J.W.C., Rajesh M.P., Tanis R.M., Cotham W.E., Frizzell N. (2014). Identification of protein succination as a novel modification of tubulin. Biochem. J..

[141] Ji S., Kang J.G., Park S.Y., Lee J., Oh Y.J., Cho J.W. (2010). O-GlcNAcylation of tubulin inhibits its polymerization. Amino Acids.

[142] Verhey K.J., Gaertig J. (2007). The Tubulin Code. Cell Cycle.

[143] Bär J., Popp Y., Bucher M., Mikhaylova M. (2022). Direct and indirect effects of tubulin post-translational modifications on microtubule stability: Insights and regulations. Biochim. Biophys. Acta (BBA)-Mol. Cell Res..

[144] Kumar N., Flavin M. (1981). Preferential action of a brain detyrosinolating carboxypeptidase on polymerized tubulin. J. Biol. Chem..

[145] Shida T., Cueva J.G., Xu Z., Goodman M.B., Nachury M.V. (2010). The major α-tubulin K40 acetyltransferase αTAT1 promotes rapid ciliogenesis and efficient mechanosensation. Proc. Natl. Acad. Sci. USA.

[146] Regnard C., Audebert S., Desbruyères É., Denoulet P., Eddé B. (1998). Tubulin Polyglutamylase: Partial Purification and Enzymatic Properties. Biochemistry.

[147] Audebert S., Desbruyères E. (1993). Reversible Polyglutamylation of Alpha- and Beta-Tubulin and Microtubule Dynamics in Mouse Brain Neurons. Mol. Biol. Cell.

[148] Zhang Y., Li N., Caron C., Matthias G., Hess D., Khochbin S., Matthias P. (2003). HDAC-6 interacts with and deacetylates tubulin and microtubules in vivo. EMBO J..

[149] Szyk A., Deaconescu A.M., Piszczek G., Roll-Mecak A. (2011). Tubulin tyrosine ligase structure reveals adaptation of an ancient fold to bind and modify tubulin. Nat. Struct. Mol. Biol..

[150] Prota A.E., Magiera M.M., Kuijpers M., Bargsten K., Frey D., Wieser M., Jaussi R., Hoogenraad C.C., Kammerer R.A., Janke C. (2013). Structural basis of tubulin tyrosination by tubulin tyrosine ligase. J. Cell Biol..

[151] He K., Ling K., Hu J. (2020). The emerging role of tubulin posttranslational modifications in cilia and ciliopathies. Biophys. Rep..

[152] Gadadhar S., Dadi H., Bodakuntla S., Schnitzler A., Bièche I., Rusconi F., Janke C. (2017). Tubulin glycylation controls primary cilia length. J. Cell Biol..

[153] Rogowski K., Juge F., van Dijk J., Wloga D., Strub J.-M., Levilliers N., Thomas D., Bré M.-H., Van Dorsselaer A., Gaertig J. (2009). Evolutionary Divergence of Enzymatic Mechanisms for Posttranslational Polyglycylation. Cell.

[154] Wloga D., Webster D.M., Rogowski K., Bré M.-H., Levilliers N., Jerka-Dziadosz M., Janke C., Dougan S.T., Gaertig J. (2009). TTLL3 Is a Tubulin Glycine Ligase that Regulates the Assembly of Cilia. Dev. Cell.

[155] Koenning M., Wang X., Karki M., Jangid R.K., Kearns S., Tripathi D.N., Cianfrocco M., Verhey K.J., Jung S.Y., Coarfa C. (2021). Neuronal SETD2 activity links microtubule methylation to an anxiety-like phenotype in mice. Brain.

[156] Xie X., Wang S., Li M., Diao L., Pan X., Chen J., Zou W., Zhang X., Feng W., Bao L. (2021). α-TubK40me3 is required for neuronal polarization and migration by promoting microtubule formation. Nat. Commun..

[157] Xu Z., Schaedel L., Portran D., Aguilar A., Gaillard J., Marinkovich M.P., Théry M., Nachury M.V. (2017). Microtubules acquire resistance from mechanical breakage through intralumenal acetylation. Science.

[158] Martínez-Hernández J., Parato J., Sharma A., Soleilhac J.-M., Qu X., Tein E., Sproul A., Andrieux A., Goldberg Y., Moutin M.-J. (2022). Crosstalk between acetylation and the tyrosination/detyrosination cycle of α-tubulin in Alzheimer’s disease. Front. Cell Dev. Biol..

[159] Rogowski K., van Dijk J., Magiera M.M., Bosc C., Deloulme J.-C., Bosson A., Peris L., Gold N.D., Lacroix B., Grau M.B. (2010). A Family of Protein-Deglutamylating Enzymes Associated with Neurodegeneration. Cell.

[160] Magiera M.M., Singh P., Gadadhar S., Janke C. (2018). Tubulin Posttranslational Modifications and Emerging Links to Human Disease. Cell.

[161] Yu I., Garnham C.P., Roll-Mecak A. (2015). Writing and Reading the Tubulin Code. J. Biol. Chem..

[162] Kubo T., Yanagisawa H.-A., Yagi T., Hirono M., Kamiya R. (2010). Tubulin Polyglutamylation Regulates Axonemal Motility by Modulating Activities of Inner-Arm Dyneins. Curr. Biol..

[163] Suryavanshi S., Eddé B., Fox L.A., Guerrero S., Hard R., Hennessey T., Kabi A., Malison D., Pennock D., Sale W.S. (2010). Tubulin Glutamylation Regulates Ciliary Motility by Altering Inner Dynein Arm Activity. Curr. Biol..

[164] Johnson K. (1998). The axonemal microtubules of the Chlamydomonas flagellum differ in tubulin isoform content. J. Cell Sci..

[165] Akella J.S., Wloga D., Kim J., Starostina N.G., Lyons-Abbott S., Morrissette N.S., Dougan S.T., Kipreos E.T., Gaertig J. (2010). MEC-17 is an α-tubulin acetyltransferase. Nature.

[166] Kim G.-W., Li L., Gorbani M., You L., Yang X.-J. (2013). Mice Lacking α-Tubulin Acetyltransferase 1 Are Viable but Display α-Tubulin Acetylation Deficiency and Dentate Gyrus Distortion. J. Biol. Chem..

[167] Kalebic N., Sorrentino S., Perlas E., Bolasco G., Martinez C., Heppenstall P.A. (2013). αTAT1 is the major α-tubulin acetyltransferase in mice. Nat. Commun..

[168] Aguilar A., Becker L., Tedeschi T., Heller S., Iomini C., Nachury M.V. (2014). α-Tubulin K40 acetylation is required for contact inhibition of proliferation and cell–substrate adhesion. Mol. Biol. Cell.

[169] Steczkiewicz K., Kinch L., Grishin N.V., Rychlewski L., Ginalski K. (2006). Eukaryotic Domain of Unknown Function DUF738 Belongs to Gcn5-related N-acetyltransferase Superfamily. Cell Cycle.

[170] Friedmann D.R., Aguilar A., Fan J., Nachury M.V., Marmorstein R. (2012). Structure of the α-tubulin acetyltransferase, αTAT1, and implications for tubulin-specific acetylation. Proc. Natl. Acad. Sci. USA.

[171] Kormendi V., Szyk A., Piszczek G., Roll-Mecak A. (2012). Crystal Structures of Tubulin Acetyltransferase Reveal a Conserved Catalytic Core and the Plasticity of the Essential N Terminus. J. Biol. Chem..

[172] Li W., Zhong C., Li L., Sun B., Wang W., Xu S., Zhang T., Wang C., Bao L., Ding J. (2012). Molecular basis of the acetyltransferase activity of MEC-17 towards α-tubulin. Cell Res..

[173] Taschner M., Vetter M., Lorentzen E. (2012). Atomic resolution structure of human α-tubulin acetyltransferase bound to acetyl-CoA. Proc. Natl. Acad. Sci. USA.

[174] Vetting M.W., de Carvalho L.P.S., Yu M., Hegde S.S., Magnet S., Roderick S.L., Blanchard J.S. (2004). Structure and functions of the GNAT superfamily of acetyltransferases. Arch. Biochem. Biophys..

[175] Saunders H.A., Johnson-Schlitz D.M., Jenkins B.V., Volkert P.J., Yang S.Z., Wildonger J. (2022). Acetylated α-tubulin K394 regulates microtubule stability to shape the growth of axon terminals. Curr. Biol..

[176] Chu C.-W., Hou F., Zhang J., Phu L., Loktev A.V., Kirkpatrick D., Jackson P.K., Zhao Y., Zou H. (2011). A novel acetylation of β-tubulin by San modulates microtubule polymerization via down-regulating tubulin incorporation. Mol. Biol. Cell.

[177] Davenport A.M., Collins L.N., Chiu H., Minor P.J., Sternberg P.W., Hoelz A. (2014). Structural and Functional Characterization of the α-Tubulin Acetyltransferase MEC-17. J. Mol. Biol..

[178] Ohkawa N., Sugisaki S., Tokunaga E., Fujitani K., Hayasaka T., Setou M., Inokuchi K. (2008). N-acetyltransferase ARD1-NAT1 regulates neuronal dendritic development. Genes Cells.

[179] Conacci-Sorrell M., Ngouenet C., Eisenman R.N. (2010). Myc-Nick: A Cytoplasmic Cleavage Product of Myc that Promotes α-Tubulin Acetylation and Cell Differentiation. Cell.

[180] Creppe C., Malinouskaya L., Volvert M.-L., Gillard M., Close P., Malaise O., Laguesse S., Cornez I., Rahmouni S., Ormenese S. (2009). Elongator Controls the Migration and Differentiation of Cortical Neurons through Acetylation of α-Tubulin. Cell.

[181] Shen Q., Zheng X., McNutt M.A., Guang L., Sun Y., Wang J., Gong Y., Hou L., Zhang B. (2009). NAT10, a nucleolar protein, localizes to the midbody and regulates cytokinesis and acetylation of microtubules. Exp. Cell Res..

[182] Williams B.C., Garrett-Engele C.M., Li Z., Williams E.V., Rosenman E.D., Goldberg M.L. (2003). Two Putative Acetyltransferases, San and Deco, Are Required for Establishing Sister Chromatid Cohesion in *Drosophila*. Curr. Biol..

[183] Hou F., Chu C.-W., Kong X., Yokomori K., Zou H. (2007). The acetyltransferase activity of San stabilizes the mitotic cohesin at the centromeres in a shugoshin-independent manner. J. Cell Biol..

[184] Gaertig J., A Cruz M., Bowen J., Gu L., Pennock D.G., Gorovsky M.A. (1995). Acetylation of lysine 40 in alpha-tubulin is not essential in *Tetrahymena* thermophila. J. Cell Biol..

[185] Varberg J.M., Padgett L.R., Arrizabalaga G., Sullivan W.J. (2016). TgATAT-Mediated α-Tubulin Acetylation Is Required for Division of the Protozoan Parasite Toxoplasma gondii. Msphere.

[186] Castro-Castro A., Janke C., Montagnac G., Paul-Gilloteaux P., Chavrier P. (2012). ATAT1/MEC-17 acetyltransferase and HDAC6 deacetylase control a balance of acetylation of alpha-tubulin and cortactin and regulate MT1-MMP trafficking and breast tumor cell invasion. Eur. J. Cell Biol..

[187] Kalebic N., Martinez C., Perlas E., Hublitz P., Bilbao-Cortes D., Fiedorczuk K., Andolfo A., Heppenstall P.A. (2013). Tubulin Acetyltransferase αTAT1 Destabilizes Microtubules Independently of Its Acetylation Activity. Mol. Cell. Biol..

[188] Chalfie M., Au M. (1989). Genetic Control of Differentiation of the *Caenorhabditis elegans* Touch Receptor Neurons. Science.

[189] Zhang Y., Ma C., Delohery T., Nasipak B., Foat B.C., Bounoutas A., Bussemaker H.J., Kim S.K., Chalfie M. (2002). Identification of genes expressed in *C. elegans* touch receptor neurons. Nature.

[190] Topalidou I., Keller C., Kalebic N., Nguyen K.C., Somhegyi H., Politi K.A., Heppenstall P., Hall D.H., Chalfie M. (2012). Genetically Separable Functions of the MEC-17 Tubulin Acetyltransferase Affect Microtubule Organization. Curr. Biol..

[191] Zhang K., Wang D., Song J. (2009). Cortactin is involved in transforming growth factor-β1-induced epithelial-mesenchymal transition in AML-12 cells. Acta Biochim. Biophys. Sin..

[192] Montagnac G., Meas-Yedid V., Irondelle M., Castro-Castro A., Franco M., Shida T., Nachury M.V., Benmerah A., Olivo-Marin J.-C., Chavrier P. (2013). αTAT1 catalyses microtubule acetylation at clathrin-coated pits. Nature.

[193] Nogales E., Whittaker M., Milligan R.A., Downing K.H. (1999). High-Resolution Model of the Microtubule. Cell.

[194] Soppina V., Herbstman J.F., Skiniotis G., Verhey K.J. (2012). Luminal Localization of α-tubulin K40 Acetylation by Cryo-EM Analysis of Fab-Labeled Microtubules. PLoS ONE.

[195] Szyk A., Deaconescu A.M., Spector J., Goodman B., Valenstein M.L., Ziolkowska N.E., Kormendi V., Grigorieff N., Roll-Mecak A. (2014). Molecular Basis for Age-Dependent Microtubule Acetylation by Tubulin Acetyltransferase. Cell.

[196] Coombes C., Yamamoto A., McClellan M., Reid T.A., Plooster M., Luxton G.W.G., Alper J., Howard J., Gardner M.K. (2016). Mechanism of microtubule lumen entry for the α-tubulin acetyltransferase enzyme αTAT1. Proc. Natl. Acad. Sci. USA.

[197] Ly N., Elkhatib N., Bresteau E., Piétrement O., Khaled M., Magiera M.M., Janke C., Le Cam E., Rutenberg A.D., Montagnac G. (2016). αTAT1 controls longitudinal spreading of acetylation marks from open microtubules extremities. Sci. Rep..

[198] Odde D. (1998). Diffusion inside microtubules. Eur. Biophys. J..

[199] Akhmanova A., Steinmetz M.O. (2015). Control of microtubule organization and dynamics: Two ends in the limelight. Nat. Rev. Mol. Cell Biol..

[200] Chrétien D., Metoz F., Verde F., Karsenti E., Wade R.H. (1992). Lattice defects in microtubules: Protofilament numbers vary within individual microtubules. J. Cell Biol..

[201] Schaedel L., John K., Gaillard J., Nachury M.V., Blanchoin L., Théry M. (2015). Microtubules self-repair in response to mechanical stress. Nat. Mater..

[202] Howes S.C., Alushin G.M., Shida T., Nachury M.V., Nogales E. (2014). Effects of tubulin acetylation and tubulin acetyltransferase binding on microtubule structure. Mol. Biol. Cell.

[203] Janke C., Montagnac G. (2017). Causes and Consequences of Microtubule Acetylation. Curr. Biol..

[204] Hubbert C., Guardiola A., Shao R., Kawaguchi Y., Ito A., Nixon A., Yoshida M., Wang X.-F., Yao T.-P. (2002). HDAC6 is a microtubule-associated deacetylase. Nature.

[205] Zhang Y., Kwon S., Yamaguchi T., Cubizolles F., Rousseaux S., Kneissel M., Cao C., Li N., Cheng H.-L., Chua K. (2008). Mice Lacking Histone Deacetylase 6 Have Hyperacetylated Tubulin but Are Viable and Develop Normally. Mol. Cell. Biol..

[206] North B.J., Marshall B.L., Borra M.T., Denu J.M., Verdin E. (2003). The Human Sir2 Ortholog, SIRT2, Is an NAD+-Dependent Tubulin Deacetylase. Mol. Cell.

[207] Toro T.B., Watt T.J. (2020). Critical review of non-histone human substrates of metal-dependent lysine deacetylases. FASEB J..

[208] Yao Y.-L., Yang W.-M. (2010). Beyond Histone and Deacetylase: An Overview of Cytoplasmic Histone Deacetylases and Their Nonhistone Substrates. J. Biomed. Biotechnol..

[209] Vaquero A., Scher M.B., Lee D.H., Sutton A., Cheng H.-L., Alt F.W., Serrano L., Sternglanz R., Reinberg D. (2006). SirT2 is a histone deacetylase with preference for histone H4 Lys 16 during mitosis. Genes Dev..

[210] North B.J., Verdin E. (2007). Interphase Nucleo-Cytoplasmic Shuttling and Localization of SIRT2 during Mitosis. PLoS ONE.

[211] Dryden S.C., Nahhas F.A., Nowak J.E., Goustin A.-S., Tainsky M.A. (2003). Role for Human SIRT2 NAD-Dependent Deacetylase Activity in Control of Mitotic Exit in the Cell Cycle. Mol. Cell. Biol..

[212] Kim H.-S., Vassilopoulos A., Wang R.-H., Lahusen T., Xiao Z., Xu X., Li C., Veenstra T.D., Li B., Yu H. (2011). SIRT2 Maintains Genome Integrity and Suppresses Tumorigenesis through Regulating APC/C Activity. Cancer Cell.

[213] Zhang H., Park S.-H., Pantazides B.G., Karpiuk O., Warren M.D., Hardy C.W., Duong D.M., Park S.-J., Kim H.-S., Vassilopoulos A. (2013). SIRT2 directs the replication stress response through CDK9 deacetylation. Proc. Natl. Acad. Sci. USA.

[214] Pandithage R., Lilischkis R., Harting K., Wolf A., Jedamzik B., Lüscher-Firzlaff J., Vervoorts J., Lasonder E., Kremmer E., Knöll B. (2008). The regulation of SIRT2 function by cyclin-dependent kinases affects cell motility. J. Cell Biol..

[215] de Oliveira R.M., Vicente Miranda H., Francelle L., Pinho R., Szegö É.M., Martinho R., Munari F., Lázaro D.F., Moniot S., Guerreiro P. (2017). The mechanism of sirtuin 2–mediated exacerbation of alpha-synuclein toxicity in models of Parkinson disease. PLoS Biol..

[216] Quinti L., Casale M., Moniot S., Pais T.F., Van Kanegan M.J., Kaltenbach L.S., Pallos J., Lim R.G., Naidu S.D., Runne H. (2016). SIRT2- and NRF2-Targeting Thiazole-Containing Compound with Therapeutic Activity in Huntington’s Disease Models. Cell Chem. Biol..

[217] Biella G., Fusco F., Nardo E., Bernocchi O., Colombo A., Lichtenthaler S.F., Forloni G., Albani D. (2016). Sirtuin 2 Inhibition Improves Cognitive Performance and Acts on Amyloid-β Protein Precursor Processing in Two Alzheimer’s Disease Mouse Models. J. Alzheimer’s Dis..

[218] Garske A.L., Smith B.C., Denu J.M. (2007). Linking SIRT2 to Parkinson’s Disease. ACS Chem. Biol..

[219] Liu Y., Peng L., Seto E., Huang S., Qiu Y. (2012). Modulation of Histone Deacetylase 6 (HDAC6) Nuclear Import and Tubulin Deacetylase Activity through Acetylation. J. Biol. Chem..

[220] Li Y., Shin D., Kwon S.H. (2012). Histone deacetylase 6 plays a role as a distinct regulator of diverse cellular processes. FEBS J..

[221] Fusco C., Micale L., Augello B., Mandriani B., Pellico M.T., De Nittis P., Calcagnì A., Monti M., Cozzolino F., Pucci P. (2014). HDAC6 mediates the acetylation of TRIM50. Cell. Signal..

[222] Kawaguchi Y., Kovacs J.J., McLaurin A., Vance J.M., Ito A., Yao T.-P. (2003). The Deacetylase HDAC6 Regulates Aggresome Formation and Cell Viability in Response to Misfolded Protein Stress. Cell.

[223] Boyault C., Sadoul K., Pabion M., Khochbin S. (2007). HDAC6, at the crossroads between cytoskeleton and cell signaling by acetylation and ubiquitination. Oncogene.

[224] Chen S., Owens G.C., Makarenkova H., Edelman D.B. (2010). HDAC6 Regulates Mitochondrial Transport in Hippocampal Neurons. PLoS ONE.

[225] Simões-Pires C., Zwick V., Nurisso A., Schenker E., Carrupt P.-A., Cuendet M. (2013). HDAC6 as a target for neurodegenerative diseases: What makes it different from the other HDACs?. Mol. Neurodegener..

[226] Shen S., Svoboda M., Zhang G., Cavasin M.A., Motlová L., McKinsey T.A., Eubanks J.H., Bařinka C., Kozikowski A.P. (2020). Structural and in Vivo Characterization of Tubastatin A, a Widely Used Histone Deacetylase 6 Inhibitor. ACS Med. Chem. Lett..

[227] Li T., Zhang C., Hassan S., Liu X., Song F., Chen K., Zhang W., Yang J. (2018). Histone deacetylase 6 in cancer. J. Hematol. Oncol..

[228] Seigneurin-Berny D., Verdel A., Curtet S., Lemercier C., Garin J., Rousseaux S., Khochbin S. (2001). Identification of Components of the Murine Histone Deacetylase 6 Complex: Link between Acetylation and Ubiquitination Signaling Pathways. Mol. Cell. Biol..

[229] Boyault C., Zhang Y., Fritah S., Caron C., Gilquin B., Kwon S.H., Garrido C., Yao T.-P., Vourc’H C., Matthias P. (2007). HDAC6 controls major cell response pathways to cytotoxic accumulation of protein aggregates. Genes Dev..

[230] Piperno G., Ledizet M., Chang X.J. (1987). Microtubules containing acetylated alpha-tubulin in mammalian cells in culture. J. Cell Biol..

[231] Matsuyama A., Shimazu T., Sumida Y., Saito A., Yoshimatsu Y., Seigneurin-Berny D., Osada H., Komatsu Y., Nishino N., Khochbin S. (2002). In vivo destabilization of dynamic microtubules by HDAC6-mediated deacetylation. EMBO J..

[232] Zhao Z., Xu H., Gong W. (2010). Histone Deacetylase 6 (HDAC6) Is an Independent Deacetylase for α-Tubulin. Protein Pept. Lett..

[233] Miyake Y., Keusch J.J., Wang L., Saito M., Hess D., Wang X., Melancon B.J., Helquist P., Gut H., Matthias P. (2016). Structural insights into HDAC6 tubulin deacetylation and its selective inhibition. Nat. Chem. Biol..

[234] Zilberman Y., Ballestrem C., Carramusa L., Mazitschek R., Khochbin S., Bershadsky A. (2009). Regulation of microtubule dynamics by inhibition of the tubulin deacetylase HDAC6. J. Cell Sci..

[235] Bobrowska A., Donmez G., Weiss A., Guarente L.P., Bates G.P. (2012). SIRT2 Ablation Has No Effect on Tubulin Acetylation in Brain, Cholesterol Biosynthesis or the Progression of Huntington’s Disease Phenotypes In Vivo. PLoS ONE.

[236] Skoge R.H., Ziegler M. (2016). SIRT2 inactivation reveals a subset of hyperacetylated perinuclear microtubules inaccessible to HDAC6. J. Cell Sci..

[237] Tran A., Marmo T.P., Salam A.A., Che S., Finkelstein E., Kabarriti R., Xenias H.S., Mazitschek R., Hubbert C., Kawaguchi Y. (2007). HDAC6 deacetylation of tubulin modulates dynamics of cellular adhesions. J. Cell Sci..

[238] Cabrero J.R., Serrador J.M., Barreiro O., Mittelbrunn M., Naranjo-Suárez S., Martin-Cofreces N., Vicente-Manzanares M., Mazitschek R., Bradner J.E., Ávila J. (2006). Lymphocyte Chemotaxis Is Regulated by Histone Deacetylase 6, Independently of Its Deacetylase Activity. Mol. Biol. Cell.

[239] Zhang X., Yuan Z., Zhang Y., Yong S., Salas-Burgos A., Koomen J., Olashaw N., Parsons J.T., Yang X.-J., Dent S.R. (2007). HDAC6 Modulates Cell Motility by Altering the Acetylation Level of Cortactin. Mol. Cell.

[240] Pugacheva E.N., Jablonski S.A., Hartman T.R., Henske E.P., Golemis E.A. (2007). HEF1-Dependent Aurora A Activation Induces Disassembly of the Primary Cilium. Cell.

[241] Yang Y., Ran J., Liu M., Li D., Li Y., Shi X., Meng D., Pan J., Ou G., Aneja R. (2014). CYLD mediates ciliogenesis in multiple organs by deubiquitinating Cep70 and inactivating HDAC6. Cell Res..

[242] Zhou X., Fan L.X., Li K., Ramchandran R., Calvet J.P., Li X. (2013). SIRT2 regulates ciliogenesis and contributes to abnormal centrosome amplification caused by loss of polycystin-1. Hum. Mol. Genet..

[243] Bangs F.K., Schrode N., Hadjantonakis A.-K., Anderson K.V. (2015). Lineage specificity of primary cilia in the mouse embryo. Nat. Cell Biol..

[244] Ran J., Yang Y., Li D., Liu M., Zhou J. (2015). Deacetylation of α-tubulin and cortactin is required for HDAC6 to trigger ciliary disassembly. Sci. Rep..

[245] Liang Y., Meng D., Zhu B., Pan J. (2016). Mechanism of ciliary disassembly. Cell. Mol. Life Sci..

[246] Mackeh R., Lorin S., Ratier A., Mejdoubi-Charef N., Baillet A., Bruneel A., Hamaï A., Codogno P., Poüs C., Perdiz D. (2014). Reactive Oxygen Species, AMP-activated Protein Kinase, and the Transcription Cofactor p300 Regulate α-Tubulin Acetyltransferase-1 (αTAT-1/MEC-17)-dependent Microtubule Hyperacetylation during Cell Stress. J. Biol. Chem..

[247] Han Y., Jeong H.M., Jin Y.-H., Kim Y.-J., Jeong H.G., Yeo C.-Y., Lee K.-Y. (2009). Acetylation of histone deacetylase 6 by p300 attenuates its deacetylase activity. Biochem. Biophys. Res. Commun..

[248] Even A., Morelli G., Turchetto S., Shilian M., Le Bail R., Laguesse S., Krusy N., Brisker A., Brandis A., Inbar S. (2021). ATP-citrate lyase promotes axonal transport across species. Nat. Commun..

[249] Houtkooper R.H., Cantó C., Wanders R.J., Auwerx J. (2010). The Secret Life of NAD+: An Old Metabolite Controlling New Metabolic Signaling Pathways. Endocr. Rev..

[250] Cantó C., Menzies K.J., Auwerx J. (2015). NAD+ Metabolism and the Control of Energy Homeostasis: A Balancing Act between Mitochondria and the Nucleus. Cell Metab..

[251] Covarrubias A.J., Perrone R., Grozio A., Verdin E. (2020). NAD+ metabolism and its roles in cellular processes during ageing. Nat. Rev. Mol. Cell Biol..

[252] Shi L., Tu B.P. (2015). Acetyl-CoA and the regulation of metabolism: Mechanisms and consequences. Curr. Opin. Cell Biol..

[253] Piperno G., Fuller M.T. (1985). Monoclonal antibodies specific for an acetylated form of alpha-tubulin recognize the antigen in cilia and flagella from a variety of organisms. J. Cell Biol..

[254] LeDizet M., Piperno G. (1987). Identification of an acetylation site of Chlamydomonas alpha-tubulin. Proc. Natl. Acad. Sci. USA.

[255] Kull F.J., Sloboda R.D. (2014). A Slow Dance for Microtubule Acetylation. Cell.

[256] Maruta H., Greer K., Rosenbaum J.L. (1986). The acetylation of alpha-tubulin and its relationship to the assembly and disassembly of microtubules. J. Cell Biol..

[257] Palazzo A., Ackerman B., Gundersen G.G. (2003). Tubulin acetylation and cell motility. Nature.

[258] Webster D., Borisy G. (1989). Microtubules are acetylated in domains that turn over slowly. J. Cell Sci..

[259] Haggarty S.J., Koeller K.M., Wong J.C., Grozinger C.M., Schreiber S.L. (2003). Domain-selective small-molecule inhibitor of histone deacetylase 6 (HDAC6)-mediated tubulin deacetylation. Proc. Natl. Acad. Sci. USA.

[260] Cueva J.G., Hsin J., Huang K.C., Goodman M.B. (2012). Posttranslational Acetylation of α-Tubulin Constrains Protofilament Number in Native Microtubules. Curr. Biol..

[261] Chrétien D., Flyvbjerg H., Fuller S.D. (1998). Limited flexibility of the inter-protofilament bonds in microtubules assembled from pure tubulin. Eur. Biophys. J..

[262] Wang C., Guo Z., Wang R., Luo Y. (2016). Role of the inter-protofilament sliding in the bending of protein microtubules. J. Biomech..

[263] Eshun-Wilson L., Zhang R., Portran D., Nachury M.V., Toso D.B., Löhr T., Vendruscolo M., Bonomi M., Fraser J.S., Nogales E. (2019). Effects of α-tubulin acetylation on microtubule structure and stability. Proc. Natl. Acad. Sci. USA.

[264] Portran D., Schaedel L., Xu Z., Théry M., Nachury M.V. (2017). Tubulin acetylation protects long-lived microtubules against mechanical ageing. Nature.

[265] Neumann B., Hilliard M.A. (2014). Loss of MEC-17 Leads to Microtubule Instability and Axonal Degeneration. Cell Rep..

[266] Moutin M., Bosc C., Peris L., Andrieux A. (2020). Tubulin post-translational modifications control neuronal development and functions. Dev. Neurobiol..

[267] Mao C.-X., Xiong Y., Xiong Z., Wang Q., Zhang Y.Q., Jin S. (2014). Microtubule-severing protein Katanin regulates neuromuscular junction development and dendritic elaboration in *Drosophila*. Development.

[268] Sudo H., Baas P.W. (2010). Acetylation of Microtubules Influences Their Sensitivity to Severing by Katanin in Neurons and Fibroblasts. J. Neurosci..

[269] Leo L., Yu W., D’Rozario M., Waddell E.A., Marenda D.R., Baird M.A., Davidson M.W., Zhou B., Wu B., Baker L. (2015). Vertebrate Fidgetin Restrains Axonal Growth by Severing Labile Domains of Microtubules. Cell Rep..

[270] Nekooki-Machida Y., Nakakura T., Nishijima Y., Tanaka H., Arisawa K., Kiuchi Y., Miyashita T., Hagiwara H. (2018). Dynamic localization of α-tubulin acetyltransferase ATAT1 through the cell cycle in human fibroblastic KD cells. Med. Mol. Morphol..

[271] L’Hernault S.W., Rosenbaum J.L. (1985). Chlamydomonas. alpha.-tubulin is posttranslationally modified by acetylation on the.epsilon.-amino group of a lysine. Biochemistry.

[272] Nakakura T., Suzuki T., Nemoto T., Tanaka H., Asano-Hoshino A., Arisawa K., Nishijima Y., Kiuchi Y., Hagiwara H. (2015). Intracellular localization of α-tubulin acetyltransferase ATAT1 in rat ciliated cells. Med. Mol. Morphol..

[273] Nakakura T., Asano-Hoshino A., Suzuki T., Arisawa K., Tanaka H., Sekino Y., Kiuchi Y., Kawai K., Hagiwara H. (2014). The elongation of primary cilia via the acetylation of α-tubulin by the treatment with lithium chloride in human fibroblast KD cells. Med. Mol. Morphol..

[274] Keryer G., Pineda J.R., Liot G., Kim J., Dietrich P., Benstaali C., Smith K., Cordelières F., Spassky N., Ferrante R.J. (2011). Ciliogenesis is regulated by a huntingtin-HAP1-PCM1 pathway and is altered in Huntington disease. J. Clin. Investig..

[275] Loktev A.V., Zhang Q., Beck J.S., Searby C.C., Scheetz T.E., Bazan J.F., Slusarski D.C., Sheffield V.C., Jackson P.K., Nachury M.V. (2008). A BBSome Subunit Links Ciliogenesis, Microtubule Stability, and Acetylation. Dev. Cell.

[276] Kozminski K.G., Diener D.R., Rosenbaum J.L. (1993). High level expression of nonacetylatable ?-tubulin inChlamydomonas reinhardtii. Cell Motil. Cytoskelet..

[277] Alper J.D., Decker F., Agana B., Howard J. (2014). The Motility of Axonemal Dynein Is Regulated by the Tubulin Code. Biophys. J..

[278] Reed N.A., Cai D., Blasius T.L., Jih G.T., Meyhofer E., Gaertig J., Verhey K.J. (2006). Microtubule Acetylation Promotes Kinesin-1 Binding and Transport. Curr. Biol..

[279] Kaul N., Soppina V., Verhey K.J. (2014). Effects of α-Tubulin K40 Acetylation and Detyrosination on Kinesin-1 Motility in a Purified System. Biophys. J..

[280] Walter W.J., Beránek V., Fischermeier E., Diez S. (2012). Tubulin Acetylation Alone Does Not Affect Kinesin-1 Velocity and Run Length In Vitro. PLoS ONE.

[281] Sullenberger C., Vasquez-Limeta A., Kong D., Loncarek J. (2020). With Age Comes Maturity: Biochemical and Structural Transformation of a Human Centriole in the Making. Cells.

[282] Guichard P., Laporte M.H., Hamel V. (2023). The centriolar tubulin code. Semin. Cell Dev. Biol..

[283] Sahabandu N., Kong D., Magidson V., Nanjundappa R., Sullenberger C., Mahjoub M., Loncarek J. (2019). Expansion microscopy for the analysis of centrioles and cilia. J. Microsc..

[284] Nekooki-Machida Y., Hagiwara H. (2020). Role of tubulin acetylation in cellular functions and diseases. Med. Mol. Morphol..

[285] Ahmad F., Pienkowski T.P., Baas P.W. (1993). Regional differences in microtubule dynamics in the axon. J. Neurosci..

[286] A Cambray-Deakin M., Burgoyne R.D. (1987). Posttranslational modifications of alpha-tubulin: Acetylated and detyrosinated forms in axons of rat cerebellum. J. Cell Biol..

[287] Brown A., Li Y., Slaughter T., Black M. (1993). Composite microtubules of the axon: Quantitative analysis of tyrosinated and acetylated tubulin along individual axonal microtubules. J. Cell Sci..

[288] Morley S.J., Qi Y., Iovino L., Andolfi L., Guo D., Kalebic N., Castaldi L., Tischer C., Portulano C., Bolasco G. (2016). Acetylated tubulin is essential for touch sensation in mice. Elife.

[289] Bounoutas A., O’Hagan R., Chalfie M. (2009). The Multipurpose 15-Protofilament Microtubules in *C. elegans* Have Specific Roles in Mechanosensation. Curr. Biol..

[290] Yan C., Wang F., Peng Y., Williams C., Jenkins B., Wildonger J., Kim H.-J., Perr J.B., Vaughan J.C., Kern M.E. (2018). Microtubule Acetylation Is Required for Mechanosensation in *Drosophila*. Cell Rep..

[291] Tas R.P., Chazeau A., Cloin B.M., Lambers M.L., Hoogenraad C.C., Kapitein L.C. (2017). Differentiation between Oppositely Oriented Microtubules Controls Polarized Neuronal Transport. Neuron.

[292] Bhuwania R., Castro-Castro A., Linder S. (2014). Microtubule acetylation regulates dynamics of KIF1C-powered vesicles and contact of microtubule plus ends with podosomes. Eur. J. Cell Biol..

[293] Cai D., McEwen D.P., Martens J.R., Meyhofer E., Verhey K.J. (2009). Single Molecule Imaging Reveals Differences in Microtubule Track Selection Between Kinesin Motors. PLOS Biol..

[294] Dompierre J.P., Godin J.D., Charrin B.C., Cordelières F.P., King S.J., Humbert S., Saudou F. (2007). Histone Deacetylase 6 Inhibition Compensates for the Transport Deficit in Huntington’s Disease by Increasing Tubulin Acetylation. J. Neurosci..

[295] Godena V.K., Brookes-Hocking N., Moller A., Shaw G., Oswald M., Sancho R.M., Miller C.C.J., Whitworth A.J., De Vos K.J. (2014). Increasing microtubule acetylation rescues axonal transport and locomotor deficits caused by LRRK2 Roc-COR domain mutations. Nat. Commun..

[296] Friedman J.R., Webster B.M., Mastronarde D.N., Verhey K.J., Voeltz G.K. (2010). ER sliding dynamics and ER-mitochondrial contacts occur on acetylated microtubules. J. Cell Biol..

[297] Misawa T., Takahama M., Kozaki T., Lee H., Zou J., Saitoh T., Akira S. (2013). Microtubule-driven spatial arrangement of mitochondria promotes activation of the NLRP3 inflammasome. Nat. Immunol..

[298] Even A., Morelli G., Broix L., Scaramuzzino C., Turchetto S., Gladwyn-Ng I., Le Bail R., Shilian M., Freeman S., Magiera M.M. (2019). ATAT1-enriched vesicles promote microtubule acetylation via axonal transport. Sci. Adv..

[299] Sadoul K., Khochbin S. (2016). The growing landscape of tubulin acetylation: Lysine 40 and many more. Biochem. J..

[300] Lessard D.V., Zinder O.J., Hotta T., Verhey K.J., Ohi R., Berger C.L. (2019). Polyglutamylation of tubulin’s C-terminal tail controls pausing and motility of kinesin-3 family member KIF1A. J. Biol. Chem..

[301] Semenova I., Ikeda K., Resaul K., Kraikivski P., Aguiar M., Gygi S., Zaliapin I., Cowan A., Rodionov V. (2014). Regulation of microtubule-based transport by MAP4. Mol. Biol. Cell.

[302] Tymanskyj S.R., Yang B.H., Verhey K.J., Ma L. (2018). MAP7 regulates axon morphogenesis by recruiting kinesin-1 to microtubules and modulating organelle transport. Elife.

[303] Monroy B.Y., Sawyer D.L., Ackermann B.E., Borden M.M., Tan T.C., Ori-McKenney K.M. (2018). Competition between microtubule-associated proteins directs motor transport. Nat. Commun..

[304] Siahaan V., Krattenmacher J., Hyman A.A., Diez S., Hernández-Vega A., Lansky Z., Braun M. (2019). Kinetically distinct phases of tau on microtubules regulate kinesin motors and severing enzymes. Nature.

[305] Takemura R., Okabe S., Umeyama T., Kanai Y., Cowan N., Hirokawa N. (1992). Increased microtubule stability and alpha tubulin acetylation in cells transfected with microtubule-associated proteins MAP1B, MAP2 or tau. J. Cell Sci..

[306] Lee G., Rook S. (1992). Expression of tau protein in non-neuronal cells: Microtubule binding and stabilization. J. Cell Sci..

[307] Saragoni L., Hernandez P., Maccioni R.B. (2000). Differential Association of Tau With Subsets of Microtubules Containing Posttranslationally-Modified Tubulin Variants in Neuroblastoma Cells. Neurochem. Res..

[308] Perez M., Santa-Maria I., de Barreda E.G., Zhu X., Cuadros R., Cabrero J.R., Sanchez-Madrid F., Dawson H.N., Vitek M.P., Perry G. (2009). Tau—An inhibitor of deacetylase HDAC6 function. J. Neurochem..

[309] Mao C.-X., Wen X., Jin S., Zhang Y.Q. (2017). Increased acetylation of microtubules rescues human tau-induced microtubule defects and neuromuscular junction abnormalities in *Drosophila*. Dis. Model. Mech..

[310] Esteves A., Palma A., Gomes R., Santos D., Silva D., Cardoso S. (2018). Acetylation as a major determinant to microtubule-dependent autophagy: Relevance to Alzheimer’s and Parkinson disease pathology. Biochim. Biophys. Acta (BBA)-Mol. Basis Dis..

[311] Barisic M., e Sousa R.S., Tripathy S.K., Magiera M.M., Zaytsev A.V., Pereira A.L., Janke C., Grishchuk E.L., Maiato H. (2015). Microtubule detyrosination guides chromosomes during mitosis. Science.

[312] Wilson P.J., Forer A. (1989). Acetylated ?-tubulin in spermatogenic cells of the crane flyNephrotoma suturalis: Kinetochore microtubules are selectively acetylated. Cell Motil. Cytoskelet..

[313] Akera T. (2023). Tubulin post-translational modifications in meiosis. Semin. Cell Dev. Biol..

[314] Schatten G., Simerly C., Asai D.J., Szöke E., Cooke P., Schatten H. (1988). Acetylated α-tubulin in microtubules during mouse fertilization and early development. Dev. Biol..

[315] Wolf K.W. (1995). Acetylation of α-tubilin in different bovine cell types: Implications for microtubule dynamics in interphase and mitosis. Cell Biol. Int..

[316] Chu D.T., Klymkowsky M. (1989). The appearance of acetylated α-tubulin during early development and cellular differentiation in Xenopus. Dev. Biol..

[317] Inoue T., Hiratsuka M., Osaki M., Oshimura M. (2007). The Molecular Biology of Mammalian SIRT Proteins: SIRT2 Functions on Cell Cycle Regulation. Cell Cycle.

[318] Nagai T., Ikeda M., Chiba S., Kanno S.-I., Mizuno K. (2013). Furry promotes acetylation of microtubules in the mitotic spindle by inhibition of SIRT2 tubulin deacetylase. J. Cell Sci..

[319] A Wickström S., Masoumi K.C., Khochbin S., Fässler R., Massoumi R. (2009). CYLD negatively regulates cell-cycle progression by inactivating HDAC6 and increasing the levels of acetylated tubulin. EMBO J..

[320] Patel-Hett S., Richardson J.L., Schulze H., Drabek K., Isaac N.A., Hoffmeister K., Shivdasani R.A., Bulinski J.C., Galjart N., Hartwig J.H. (2008). Visualization of microtubule growth in living platelets reveals a dynamic marginal band with multiple microtubules. Blood.

[321] Sadoul K., Wang J., Diagouraga B., Vitte A.-L., Buchou T., Rossini T., Polack B., Xi X., Matthias P., Khochbin S. (2012). HDAC6 controls the kinetics of platelet activation. Blood.

[322] Deakin N.O., Turner C.E. (2014). Paxillin inhibits HDAC6 to regulate microtubule acetylation, Golgi structure, and polarized migration. J. Cell Biol..

[323] Gu S., Liu Y., Zhu B., Ding K., Yao T.-P., Chen F., Zhan L., Xu P., Ehrlich M., Liang T. (2016). Loss of α-Tubulin Acetylation Is Associated with TGF-β-induced Epithelial-Mesenchymal Transition. J. Biol. Chem..

[324] Boggs A.E., Vitolo M.I., Whipple R.A., Charpentier M.S., Goloubeva O.G., Ioffe O.B., Tuttle K.C., Slovic J., Lu Y., Mills G.B. (2015). α-Tubulin Acetylation Elevated in Metastatic and Basal-like Breast Cancer Cells Promotes Microtentacle Formation, Adhesion, and Invasive Migration. Cancer Res..

[325] Bance B., Seetharaman S., Leduc C., Boëda B., Etienne-Manneville S. (2019). Microtubule acetylation but not detyrosination promotes focal adhesion dynamics and astrocyte migration. J. Cell Sci..

[326] Collins C., Kim S.K., Ventrella R., Carruzzo H.M., Wortman J.C., Han H., Suva E.E., Mitchell J.W., Yu C.C., Mitchell B.J. (2021). Tubulin acetylation promotes penetrative capacity of cells undergoing radial intercalation. Cell Rep..

[327] Seetharaman S., Vianay B., Roca V., Farrugia A.J., De Pascalis C., Boëda B., Dingli F., Loew D., Vassilopoulos S., Bershadsky A. (2021). Microtubules tune mechanosensitive cell responses. Nat. Mater..

[328] Yang J., Song C., Zhan X. (2022). The role of protein acetylation in carcinogenesis and targeted drug discovery. Front. Endocrinol..

[329] Black M.M., Keyser P. (1987). Acetylation of alpha-tubulin in cultured neurons and the induction of alpha-tubulin acetylation in PC12 cells by treatment with nerve growth factor. J. Neurosci..

[330] Knossow M., Campanacci V., Khodja L.A., Gigant B. (2020). The Mechanism of Tubulin Assembly into Microtubules: Insights from Structural Studies. iScience.

[331] Geeraert C., Ratier A., Pfisterer S.G., Perdiz D., Cantaloube I., Rouault A., Pattingre S., Proikas-Cezanne T., Codogno P., Poüs C. (2010). Starvation-induced Hyperacetylation of Tubulin Is Required for the Stimulation of Autophagy by Nutrient Deprivation. J. Biol. Chem..

[332] Xie R., Nguyen S., McKeehan W.L., Liu L. (2010). Acetylated microtubules are required for fusion of autophagosomes with lysosomes. BMC Cell Biol..

[333] Li W., Zhao Y., Chou I.-N. (1996). Nickel (Ni2+) Enhancement of α-Tubulin Acetylation in Cultured 3T3 Cells. Toxicol. Appl. Pharmacol..

[334] Giustiniani J., Daire V., Cantaloube I., Durand G., Poüs C., Perdiz D., Baillet A. (2009). Tubulin acetylation favors Hsp90 recruitment to microtubules and stimulates the signaling function of the Hsp90 clients Akt/PKB and p53. Cell. Signal..

[335] Kratzer E., Tian Y., Sarich N., Wu T., Meliton A., Leff A., Birukova A.A. (2012). Oxidative Stress Contributes to Lung Injury and Barrier Dysfunction via Microtubule Destabilization. Am. J. Respir. Cell Mol. Biol..

[336] Oakhill J.S., Steel R., Chen Z.-P., Scott J.W., Ling N., Tam S., Kemp B.E. (2011). AMPK Is a Direct Adenylate Charge-Regulated Protein Kinase. Science.

[337] Galdieri L., Gatla H., Vancurova I., Vancura A. (2016). Activation of AMP-activated Protein Kinase by Metformin Induces Protein Acetylation in Prostate and Ovarian Cancer Cells. J. Biol. Chem..

[338] Vancura A., Nagar S., Kaur P., Bu P., Bhagwat M., Vancurova I. (2018). Reciprocal Regulation of AMPK/SNF1 and Protein Acetylation. Int. J. Mol. Sci..

[339] Currais A., Huang L., Goldberg J., Petrascheck M., Ates G., Pinto-Duarte A., Shokhirev M.N., Schubert D., Maher P. (2019). Elevating acetyl-CoA levels reduces aspects of brain aging. Elife.

[340] Tarasiuk O., Miceli M., Di Domizio A., Nicolini G. (2022). AMPK and Diseases: State of the Art Regulation by AMPK-Targeting Molecules. Biology.

[341] Zmijewski J.W., Banerjee S., Bae H., Friggeri A., Lazarowski E.R., Abraham E. (2010). Exposure to Hydrogen Peroxide Induces Oxidation and Activation of AMP-activated Protein Kinase*. J. Biol. Chem..

[342] Chen Z., Shen X., Shen F., Zhong W., Wu H., Liu S., Lai J. (2013). TAK1 activates AMPK-dependent cell death pathway in hydrogen peroxide-treated cardiomyocytes, inhibited by heat shock protein-70. Mol. Cell. Biochem..

[343] Hinchy E.C., Gruszczyk A.V., Willows R., Navaratnam N., Hall A.R., Bates G., Bright T.P., Krieg T., Carling D., Murphy M.P. (2018). Mitochondria-derived ROS activate AMP-activated protein kinase (AMPK) indirectly. J. Biol. Chem..

[344] Salminen A., Kaarniranta K. (2012). AMP-activated protein kinase (AMPK) controls the aging process via an integrated signaling network. Ageing Res. Rev..

[345] Roy A.D., Gross E.G., Pillai G.S., Seetharaman S., Etienne-Manneville S., Inoue T. (2022). Non-catalytic allostery in α-TAT1 by a phospho-switch drives dynamic microtubule acetylation. J. Cell Biol..

[346] Shah N., Kumar S., Zaman N., Pan C.C., Bloodworth J.C., Lei W., Streicher J.M., Hempel N., Mythreye K., Lee N.Y. (2018). TAK1 activation of alpha-TAT1 and microtubule hyperacetylation control AKT signaling and cell growth. Nat. Commun..

[347] Yaciuk P., Moran E. (1991). Analysis with Specific Polyclonal Antiserum Indicates That the E1A-Associated 300-KDa Product Is a Stable Nuclear Phosphoprotein That Undergoes Cell Cycle Phase-Specific Modification. Mol. Cell. Biol..

[348] Yang W., Hong Y.H., Shen X.-Q., Frankowski C., Camp H.S., Leff T. (2001). Regulation of Transcription by AMP-activated Protein Kinase: Phosphorylation of P300 Blocks Its Interaction with Nuclear Receptors. J. Biol. Chem..

[349] Zhang Y., Qiu J., Wang X., Zhang Y., Xia M. (2011). AMP-Activated Protein Kinase Suppresses Endothelial Cell Inflammation Through Phosphorylation of Transcriptional Coactivator p300. Arter. Thromb. Vasc. Biol..

[350] A Caporizzo M., Chen C.Y., Prosser B.L. (2019). Cardiac microtubules in health and heart disease. Exp. Biol. Med..

[351] Mackeh R., Perdiz D., Lorin S., Codogno P., Poüs C. (2013). Autophagy and microtubules—New story, old players. J. Cell Sci..

[352] Saxton R.A., Sabatini D.M. (2017). mTOR Signaling in Growth, Metabolism, and Disease. Cell.

[353] Chun Y., Kim J. (2018). Autophagy: An Essential Degradation Program for Cellular Homeostasis and Life. Cells.

[354] Hać A., Pierzynowska K., Herman-Antosiewicz A. (2021). S6K1 Is Indispensible for Stress-Induced Microtubule Acetylation and Autophagic Flux. Cells.

[355] Hempen B., Brion J.-P. (1996). Reduction of Acetylated α-Tubulin Immunoreactivity in Neurofibrillary Tangle-bearing Neurons in Alzheimerʼs Disease. J. Neuropathol. Exp. Neurol..

[356] Silva D.F., Esteves A.R., Oliveira C.R., Cardoso S.M. (2017). Mitochondrial Metabolism Power SIRT2-Dependent Deficient Traffic Causing Alzheimer’s-Disease Related Pathology. Mol. Neurobiol..

[357] D’Ydewalle C., Krishnan J., Chiheb D.M., Van Damme P., Irobi J., Kozikowski A.P., Van den Berghe P., Timmerman V., Robberecht W., Van den Bosch L. (2011). HDAC6 inhibitors reverse axonal loss in a mouse model of mutant HSPB1–induced Charcot-Marie-Tooth disease. Nat. Med..

[358] Benoy V., Vanden Berghe P.V., Jarpe M., Van Damme P., Robberecht W., Van Den Bosch L. (2017). Development of Improved HDAC6 Inhibitors as Pharmacological Therapy for Axonal Charcot–Marie–Tooth Disease. Neurotherapeutics.

[359] Adalbert R., Kaieda A., Antoniou C., Loreto A., Yang X., Gilley J., Hoshino T., Uga K., Makhija M.T., Coleman M.P. (2019). Novel HDAC6 Inhibitors Increase Tubulin Acetylation and Rescue Axonal Transport of Mitochondria in a Model of Charcot–Marie–Tooth Type 2F. ACS Chem. Neurosci..

[360] Picci C., Wong V., Costa C., McKinnon M.C., Goldberg D.C., Swift M., Alam N.M., Prusky G.T., Shen S., Kozikowski A.P. (2020). HDAC6 inhibition promotes α-tubulin acetylation and ameliorates CMT2A peripheral neuropathy in mice. Exp. Neurol..

[361] Taes I., Timmers M., Hersmus N., Bento-Abreu A., Van Den Bosch L., Van Damme P., Auwerx J., Robberecht W. (2013). Hdac6 deletion delays disease progression in the SOD1G93A mouse model of ALS. Hum. Mol. Genet..

[362] Govindarajan N., Rao P., Burkhardt S., Sananbenesi F., Schlüter O.M., Bradke F., Lu J., Fischer A. (2012). Reducing HDAC6 ameliorates cognitive deficits in a mouse model for Alzheimer’s disease. EMBO Mol. Med..

[363] Zhang L., Liu C., Wu J., Tao J.-J., Sui X.-L., Yao Z.-G., Xu Y.-F., Huang L., Zhu H., Sheng S.-L. (2014). Tubastatin A/ACY-1215 Improves Cognition in Alzheimer’s Disease Transgenic Mice. J. Alzheimer’s Dis..

[364] Cappelletti G., Calogero A.M., Rolando C. (2021). Microtubule acetylation: A reading key to neural physiology and degeneration. Neurosci. Lett..

[365] Spillantini M.G., Schmidt M.L., Lee V.M.-Y., Trojanowski J.Q., Jakes R., Goedert M. (1997). α-Synuclein in Lewy bodies. Nature.

[366] Zimprich A. (2011). Genetics of Parkinson’s disease and essential tremor. Curr. Opin. Neurol..

[367] Alim M.A., Ma Q.-L., Takeda K., Aizawa T., Matsubara M., Nakamura M., Asada A., Saito T., Xkaji M., Yoshii M. (2004). Demonstration of a role for α-synuclein as a functional microtubule-associated protein. J. Alzheimer’s Dis..

[368] Cartelli D., Aliverti A., Barbiroli A., Santambrogio C., Ragg E.M., Casagrande F.V., Cantele F., Beltramone S., Marangon J., De Gregorio C. (2016). α-Synuclein is a Novel Microtubule Dynamase. Sci. Rep..

[369] Outeiro T.F., Kontopoulos E., Altmann S.M., Kufareva I., Strathearn K.E., Amore A.M., Volk C.B., Maxwell M.M., Rochet J.-C., McLean P.J. (2007). Sirtuin 2 Inhibitors Rescue α-Synuclein-Mediated Toxicity in Models of Parkinson’s Disease. Science.

[370] Kluss J.H., Mamais A., Cookson M.R. (2019). LRRK2 links genetic and sporadic Parkinson’s disease. Biochem. Soc. Trans..

[371] Simón-Sánchez J., Schulte C., Bras J.M., Sharma M., Gibbs J.R., Berg D., Paisan-Ruiz C., Lichtner P., Scholz S.W., Hernandez D.G. (2009). Genome-wide association study reveals genetic risk underlying Parkinson’s disease. Nat. Genet..

[372] Snead D.M., Matyszewski M., Dickey A.M., Lin Y.X., Leschziner A.E., Reck-Peterson S.L. (2022). Structural basis for Parkinson’s disease-linked LRRK2′s binding to microtubules. Nat. Struct. Mol. Biol..

[373] Law B.M.H., Spain V.A., Leinster V.H.L., Chia R., Beilina A., Cho H.J., Taymans J.-M., Urban M.K., Sancho R.M., Ramírez M.B. (2014). A Direct Interaction between Leucine-rich Repeat Kinase 2 and Specific β-Tubulin Isoforms Regulates Tubulin Acetylation. J. Biol. Chem..

[374] Leschziner A.E., Reck-Peterson S.L. (2021). Structural Biology of LRRK2 and its Interaction with Microtubules. Mov. Disord..

[375] Deniston C.K., Salogiannis J., Mathea S., Snead D.M., Lahiri I., Matyszewski M., Donosa O., Watanabe R., Böhning J., Shiau A.K. (2020). Structure of LRRK2 in Parkinson’s disease and model for microtubule interaction. Nature.

[376] Lloret A., Fuchsberger T., Giraldo E., Viña J. (2015). Molecular mechanisms linking amyloid β toxicity and Tau hyperphosphorylation in Alzheimer׳s disease. Free. Radic. Biol. Med..

[377] Ding H., Dolan P.J., Johnson G.V.W. (2008). Histone deacetylase 6 interacts with the microtubule-associated protein tau. J. Neurochem..

[378] Selenica M.-L., Benner L., Housley S.B., Manchec B., Lee D.C., Nash K.R., Kalin J., Bergman J.A., Kozikowski A., Gordon M.N. (2014). Histone deacetylase 6 inhibition improves memory and reduces total tau levels in a mouse model of tau deposition. Alzheimer’s Res. Ther..

[379] Tseng J.-H., Xie L., Song S., Xie Y., Allen L., Ajit D., Hong J.-S., Chen X., Meeker R.B., Cohen T.J. (2017). The Deacetylase HDAC6 Mediates Endogenous Neuritic Tau Pathology. Cell Rep..

[380] Onishi T., Maeda R., Terada M., Sato S., Fujii T., Ito M., Hashikami K., Kawamoto T., Tanaka M. (2021). A novel orally active HDAC6 inhibitor T-518 shows a therapeutic potential for Alzheimer’s disease and tauopathy in mice. Sci. Rep..

[381] Dixit R., Ross J.L., Goldman Y.E., Holzbaur E.L.F. (2008). Differential Regulation of Dynein and Kinesin Motor Proteins by Tau. Science.

[382] Cohen T.J., Guo J.L., Hurtado D.E., Kwong L.K., Mills I.P., Trojanowski J.Q., Lee V.M.Y. (2011). The acetylation of tau inhibits its function and promotes pathological tau aggregation. Nat. Commun..

[383] Zhang F., Su B., Wang C., Siedlak S.L., Mondragon-Rodriguez S., Lee H.-G., Wang X., Perry G., Zhu X. (2015). Posttranslational modifications of α-tubulin in alzheimer disease. Transl. Neurodegener..

[384] Saba N.F., Magliocca K.R., Kim S., Muller S., Chen Z., Owonikoko T.K., Sarlis N.J., Eggers C., Phelan V., Grist W.J. (2013). Acetylated Tubulin (AT) as a Prognostic Marker in Squamous Cell Carcinoma of the Head and Neck. Head Neck Pathol..

[385] Seeley E.S., Carrière C., Goetze T., Longnecker D.S., Korc M. (2009). Pancreatic Cancer and Precursor Pancreatic Intraepithelial Neoplasia Lesions Are Devoid of Primary Cilia. Cancer Res.

[386] Del Bufalo D., Desideri M., De Luca T., Di Martile M., Gabellini C., Monica V., Busso S., Eramo A., De Maria R., Milella M. (2014). Histone deacetylase inhibition synergistically enhances pemetrexed cytotoxicity through induction of apoptosis and autophagy in non-small cell lung cancer. Mol. Cancer.

[387] Rey M., Irondelle M., Waharte F., Lizarraga F., Chavrier P. (2011). HDAC6 is required for invadopodia activity and invasion by breast tumor cells. Eur. J. Cell Biol..

[388] Lee C.-C., Cheng Y.-C., Chang C.-Y., Lin C.-M., Chang J.-Y. (2018). Alpha-tubulin acetyltransferase/MEC-17 regulates cancer cell migration and invasion through epithelial–mesenchymal transition suppression and cell polarity disruption. Sci. Rep..

[389] Depetter Y., Geurs S., De Vreese R., Goethals S., Vandoorn E., Laevens A., Steenbrugge J., Meyer E., De Tullio P., Bracke M. (2019). Selective pharmacological inhibitors of HDAC6 reveal biochemical activity but functional tolerance in cancer models. Int. J. Cancer.

[390] Lopes D., Seabra A.L., Orr B., Maiato H. (2022). α-Tubulin detyrosination links the suppression of MCAK activity with taxol cytotoxicity. J. Cell Biol..

[391] Herczenik E., Gebbink M.F.B.G. (2008). Molecular and cellular aspects of protein misfolding and disease. FASEB J..

[392] Willis M.S., Patterson C. (2013). Proteotoxicity and Cardiac Dysfunction—Alzheimer’s Disease of the Heart?. N. Engl. J. Med..

[393] Folger A., Wang Y. (2021). The Cytotoxicity and Clearance of Mutant Huntingtin and Other Misfolded Proteins. Cells.

[394] Wang X., Osinska H., Klevitsky R., Gerdes A.M., Nieman M., Lorenz J., Hewett T., Robbins J. (2001). Expression of R120G-αB-Crystallin Causes Aberrant Desmin and αB-Crystallin Aggregation and Cardiomyopathy in Mice. Circ. Res..

[395] Maloyan A., Sayegh J., Osinska H., Chua B.H., Robbins J. (2010). Manipulation of Death Pathways in Desmin-Related Cardiomyopathy. Circ. Res..

[396] Henning R., Brundel B.J.J.M. (2017). Proteostasis in cardiac health and disease. Nat. Rev. Cardiol..

[397] Sanbe A., Osinska H., Saffitz J.E., Glabe C.G., Kayed R., Maloyan A., Robbins J. (2004). Desmin-related cardiomyopathy in transgenic mice: A cardiac amyloidosis. Proc. Natl. Acad. Sci. USA.

[398] McLendon P.M., Ferguson B.S., Osinska H., Bhuiyan S., James J., McKinsey T.A., Robbins J. (2014). Tubulin hyperacetylation is adaptive in cardiac proteotoxicity by promoting autophagy. Proc. Natl. Acad. Sci. USA.

[399] Zhang D., Wu C.-T., Qi X., Meijering R.A., Hoogstra-Berends F., Tadevosyan A., Deniz G.C., Durdu S., Akar A.R., Sibon O.C. (2014). Activation of Histone Deacetylase-6 Induces Contractile Dysfunction Through Derailment of α-Tubulin Proteostasis in Experimental and Human Atrial Fibrillation. Circulation.

[400] Naghavi M.H., Walsh D. (2017). Microtubule Regulation and Function during Virus Infection. J. Virol..

[401] Seo D., Gammon D.B. (2022). Manipulation of Host Microtubule Networks by Viral Microtubule-Associated Proteins. Viruses.

[402] Valenzuela-Fernández A., Álvarez S., Gordon M., Barrero M., Ursa A., Cabrero J.R., Fernández G., Naranjo-Suárez S., Yáñez-Mó M., Serrador J.M. (2005). Histone Deacetylase 6 Regulates Human Immunodeficiency Virus Type 1 Infection. Mol. Biol. Cell.

[403] Xuan C., Qiao W., Gao J., Liu M., Zhang X., Cao Y., Chen Q., Geng Y., Zhou J. (2007). Regulation of Microtubule Assembly and Stability by the Transactivator of Transcription Protein of Jembrana Disease Virus. J. Biol. Chem..

[404] Kannan H., Fan S., Patel D., Bossis I., Zhang Y.-J. (2009). The Hepatitis E Virus Open Reading Frame 3 Product Interacts with Microtubules and Interferes with Their Dynamics. J. Virol..

[405] Husain M., Harrod K.S. (2010). Enhanced acetylation of alpha-tubulin in influenza A virus infected epithelial cells. FEBS Lett..

[406] Zheng K., Jiang Y., He Z., Kitazato K., Wang Y. (2017). Cellular defence or viral assist: The dilemma of HDAC6. J. Gen. Virol..

[407] Husain M., Cheung C.-Y. (2014). Histone Deacetylase 6 Inhibits Influenza A Virus Release by Downregulating the Trafficking of Viral Components to the Plasma Membrane via Its Substrate, Acetylated Microtubules. J. Virol..

[408] Naranatt P.P., Krishnan H.H., Smith M.S., Chandran B. (2005). Kaposi’s Sarcoma-Associated Herpesvirus Modulates Microtubule Dynamics via RhoA-GTP-Diaphanous 2 Signaling and Utilizes the Dynein Motors To Deliver Its DNA to the Nucleus. J. Virol..

[409] Sabo Y., Walsh D., Barry D.S., Tinaztepe S., Santos K.D.L., Goff S.P., Gundersen G.G., Naghavi M.H. (2013). HIV-1 Induces the Formation of Stable Microtubules to Enhance Early Infection. Cell Host Microbe.

[410] Zhang S., Jiang Y., Cheng Q., Zhong Y., Qin Y., Chen M. (2017). Inclusion Body Fusion of Human Parainfluenza Virus Type 3 Regulated by Acetylated α-Tubulin Enhances Viral Replication. J. Virol..

[411] Liu P., Zhang S., Ma J., Jin D., Qin Y., Chen M. (2022). Vimentin inhibits α-tubulin acetylation via enhancing α-TAT1 degradation to suppress the replication of human parainfluenza virus type 3. PLoS Pathog..

[412] Glon D., Vilmen G., Perdiz D., Hernandez E., Beauclair G., Quignon F., Berlioz-Torrent C., Maréchal V., Poüs C., Lussignol M. (2022). Essential role of hyperacetylated microtubules in innate immunity escape orchestrated by the EBV-encoded BHRF1 protein. PLoS Pathog..

[413] Destaing O., Saltel F., Gilquin B., Chabadel A., Khochbin S., Ory S., Jurdic P. (2005). A novel Rho-mDia2-HDAC6 pathway controls podosome patterning through microtubule acetylation in osteoclasts. J. Cell Sci..

[414] Chabin-Brion K., Marceiller J., Perez F., Settegrana C., Drechou A., Durand G., Poüs C. (2001). The Golgi Complex Is a Microtubule-organizing Organelle. Mol. Biol. Cell.

